# 18β-Glycyrrhetinic acid suppresses allergic airway inflammation through NF-κB and Nrf2/HO-1 signaling pathways in asthma mice

**DOI:** 10.1038/s41598-022-06455-6

**Published:** 2022-02-24

**Authors:** Jianming Liu, Yanqi Xu, Minyu Yan, Yingjie Yu, Yongmei Guo

**Affiliations:** 1 School of Pharmacy, Nanchang University, Nanchang, 330031 Jiangxi Province China; 2 School of Pharmacy, Jiangxi Medical College, Shangrao, 334000 Jiangxi Province China

**Keywords:** Diseases, Medical research

## Abstract

18β-Glycyrrhetinic acid (18β-GA), the main bioactive component of Glycyrrhizae Radix, is considered a promising anti-inflammatory and antioxidant agent. Here, we evaluated the anti-inflammatory and antioxidant effects of 18β-GA in an ovalbumin (OVA)-induced asthma mouse model, and examined the role of NF-κB and Nrf2/HO-1 signaling pathways. The histopathological changes of lung tissue in mouse were assessed by histochemical staining and counting of inflammatory cells. The levels of IgE and inflammatory cytokines in the bronchoalveolar lavage fluid of mice were detected by ELISA. In OVA-induced asthmatic mice, 18β-GA treatment can significantly improve lung function and reduce lung inflammation including infiltration of inflammatory cells. In addition, 18β-GA reduced the OVA-induced NF-κB phosphorylation in lungs of mice while increasing the expression of Nrf2 and HO-1. These results indicate that 18β-GA protects OVA-induced allergic inflammation of airway by inhibiting phosphorylation of NF-κB and enhancing the Nrf2/HO-1 pathway, and serves as a potential treatment option for allergic inflammation of airway.

## Introduction

The chronic inflammatory disease asthma affects around 300 million people worldwide^1^. Asthma is a lung disorder with characteristics of inflammation and narrowing of the respiratory tracts, which involves oxidative stresses. Increased productions of pro-inflammatory cytokines, reactive oxygen species (ROS), and growth factors are key features of asthma^2,3^. Existing antioxidant systems can remove superoxide dismutase (ROS), with catalase, superoxide dismutase (SODs), and glutathione peroxidase (GSH-Px) being the common antioxidant enzymes^4^. Heme oxygenase 1 (HO-1) is a nuclear factor erythroid 2-related factor2 (Nrf2)-regulated antioxidant that promotes the production of antioxidant molecules^5^. The HO-1 expression reduces nuclear factor kappa B (NF-κB) level, thereby inhibiting the inducible nitric oxide synthase (iNOS), and showing anti-asthmatic effects through inhibition on ROS production and inflammatory response^6^. Furthermore, a growing number of evidence suggest that NF-κB, being a multicellular transcription factor, regulates immune and inflammatory responses through modulating cytokine productions^7,8^. It is reported that in allergic asthma, the NF-κB pathway is activated, and the suppression of NF-κB signaling can relieve ovalbumin (OVA)-induced asthma^9,10^. Therefore, in the treatment of asthma, in addition to alleviating airway inflammation and obstruction, we should also consider suppressing oxidative stresses.

Currently, there are few anti-asthmatic drugs with stable curative effects and few side effects available to patients. Long-acting beta-agonist and inhaled corticosteroids are considered the most common therapeutic options to treat asthma. Although they reduce airway inflammation and attenuate respiratory symptoms, have poor responses to corticosteroid-based medications are encountered in some patients, with serious adverse effects in some cases^11,12^. The number of reported asthma cases is increasing rapidly, and alternative therapeutic approaches are urgently needed. In view of the production of ROS and other oxidants in asthma, we suggest that therapeutics targeting redox stresses and signaling molecules may effectively treat asthma^13,14^.

**Figure 1:**
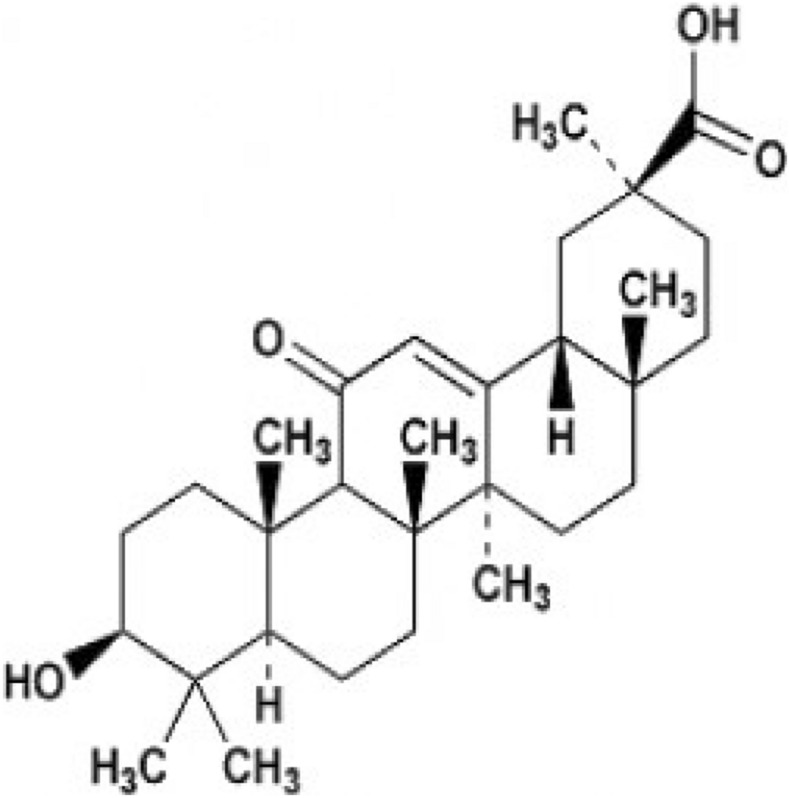
Chemical structure of 18β-GA.

Glycyrrhizic acid is the triterpene component extracted from licorice root, and in China and other Asian countries, it has been long used for the treatment of chronic hepatitis B^15^. The glycyrrhizin metabolized by intestinal flora, and its product is 18β-Glycyrrhetinic acid (18β-GA)^16^. 18β-GA showed anti-ulcer and anti-inflammation effects^17^, and its chemical structure is depicted in Fig. 1. Although 18β-GA is related to the immunoregulatory function of allergic diseases and the improvement of allergic asthma^18^, the pathophysiological role of 18β-GA in allergic lung inflammation remains unclear. Its therapeutic effect on allergic asthma and the possible mechanisms need to be further investigated. The purpose of the study is to investigate the effects of 18β-GA in treating chronic allergic asthma and its possible mechanisms using a mouse model of allergic asthma.

## Results

### 18β-GA reduces airway responsiveness of mice to methacholine (MCH) after allergen challenge

**Figure 2:**
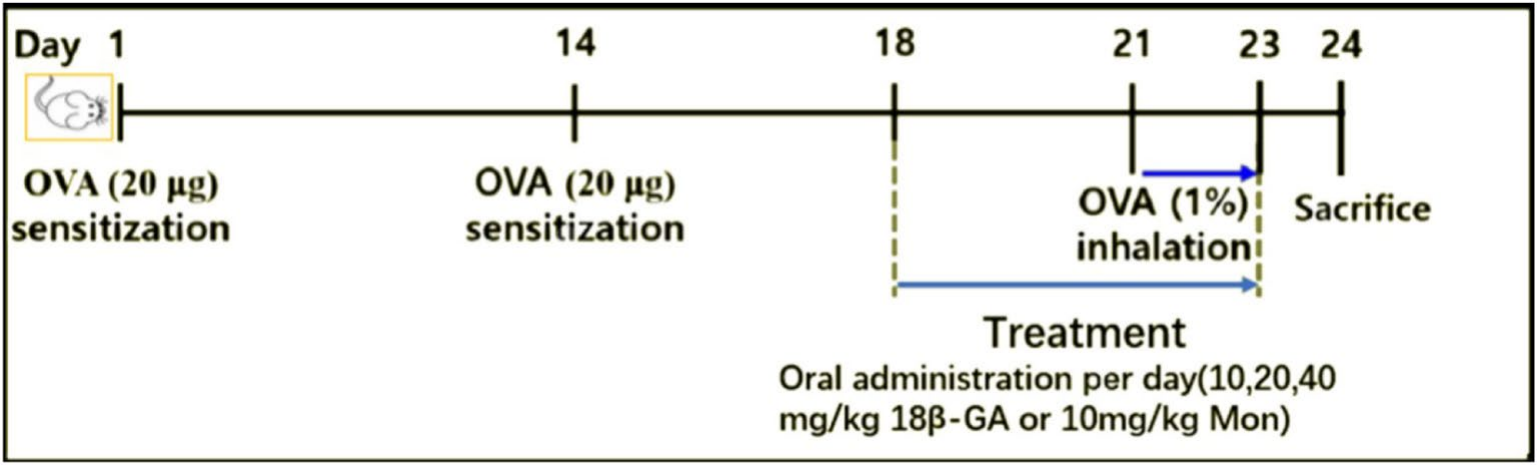
Asthmatic model and treatment protocol.

**Figure 3:**
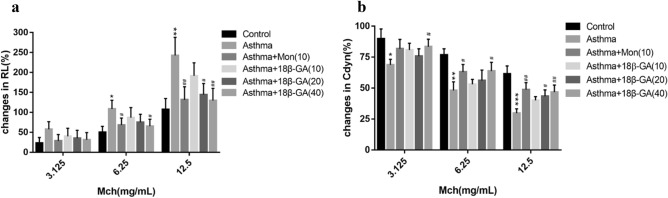
Effect of 18β-GA treatments on lung function in murine asthmatic model. (**a**) Increase of lung resistance (RL%) and (**b**) decrease of lung dynamic compliance (Cydn%). Data are shown as the mean ± SD (n = 6). *p < 0.05, **p < 0.01, ***p < 0.001 *vs*. control group, ^#^*p* < 0.05, ^##^*p* < 0.01, ^###^*p* < 0.001 *vs*. asthma group.

In order to investigate the effects of 18β-GA on asthma, OVA was used to challenge mice intranasally to establish the mouse asthmatic model to evaluate the airway response to inhaled MCH (The procedure of the experiment is presented in Fig. 2). The changes in lung resistance (RL) and lung dynamic compliance (Cdyn) were measured by increasing inhaled MCH. Compared with normal mice, the RL increased in the asthmatic mouse groups, and the RL also increased with the increase of inhaled MCH. However, the RL of asthmatic mice treated with Mon decreased significantly (Fig. 3a). The RL of asthmatic mice treated with 18β-GA also showed a downward trend, and the decrease was more obvious as the concentration of 18β-GA increased. In addition, compared with normal mice, the Cdyn in the asthmatic mouse groups decreased significantly, but Cydn increased after treatment with Mon or 18β-GA, and the increase in the Cydn was more obvious with the increase of the concentration of 18β-GA (Fig. 3b).

### 18β-GA alleviates lung pathological changes

**Figure 4:**
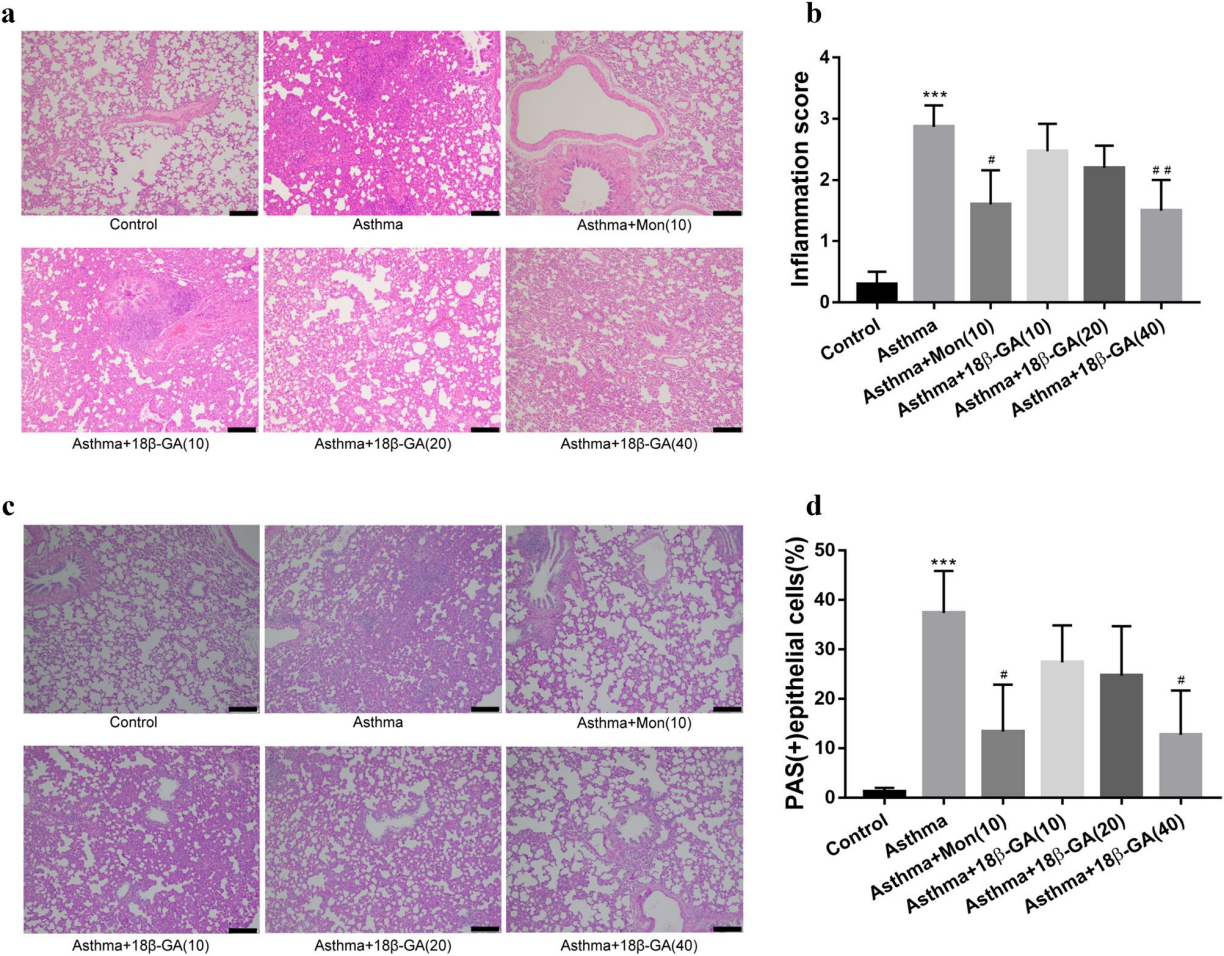
Effects of 18β-GA on OVA-induced airway inflammation and remodeling in lung tissue. (**a**) Lung sections were stained with H&E for measurement of inflammatorycells, (**c**) PAS for measurement of mucus production around the airways. × 100 magnification, scale bar: 100 μm. (**b**, **d**) The column chart of H&E and PAS were represented the mean ± SD (n = 6). *p < 0.05, **p < 0.01, ***p < 0.001 *vs*. control group, ^#^*p* < 0.05, ^##^*p* < 0.01, ^###^*p* < 0.001 *vs*. asthma group.

H&E and periodate Schiff (PAS) staining were used to detect pathological changes in the lungs. There were many infiltrated inflammatory cells in the asthma group, showing obvious vascular edema, while treatment of montelukast (Mon) or 18β-GA can significantly reduce the number of infiltrated inflammatory cells(*p* = 0.016 and *p* = 0.009, respectively)(Fig. 4a,b). The results of PAS staining showed that compared with the normal mice, the asthma mice had significantly increased mucus secretion and number of goblet cells, while both montelukast (Mon) and 18β-GA(40) could reduce the amount of mucus and the number of goblet cells(*p* = 0.037 and *p* = 0.039, respectively) (Fig. 4c,d).

### 18β-GA decreased IL-4, IL-5, IL-13, TNF-α and IgE levels while increased IFN-γ level

**Figure 5:**
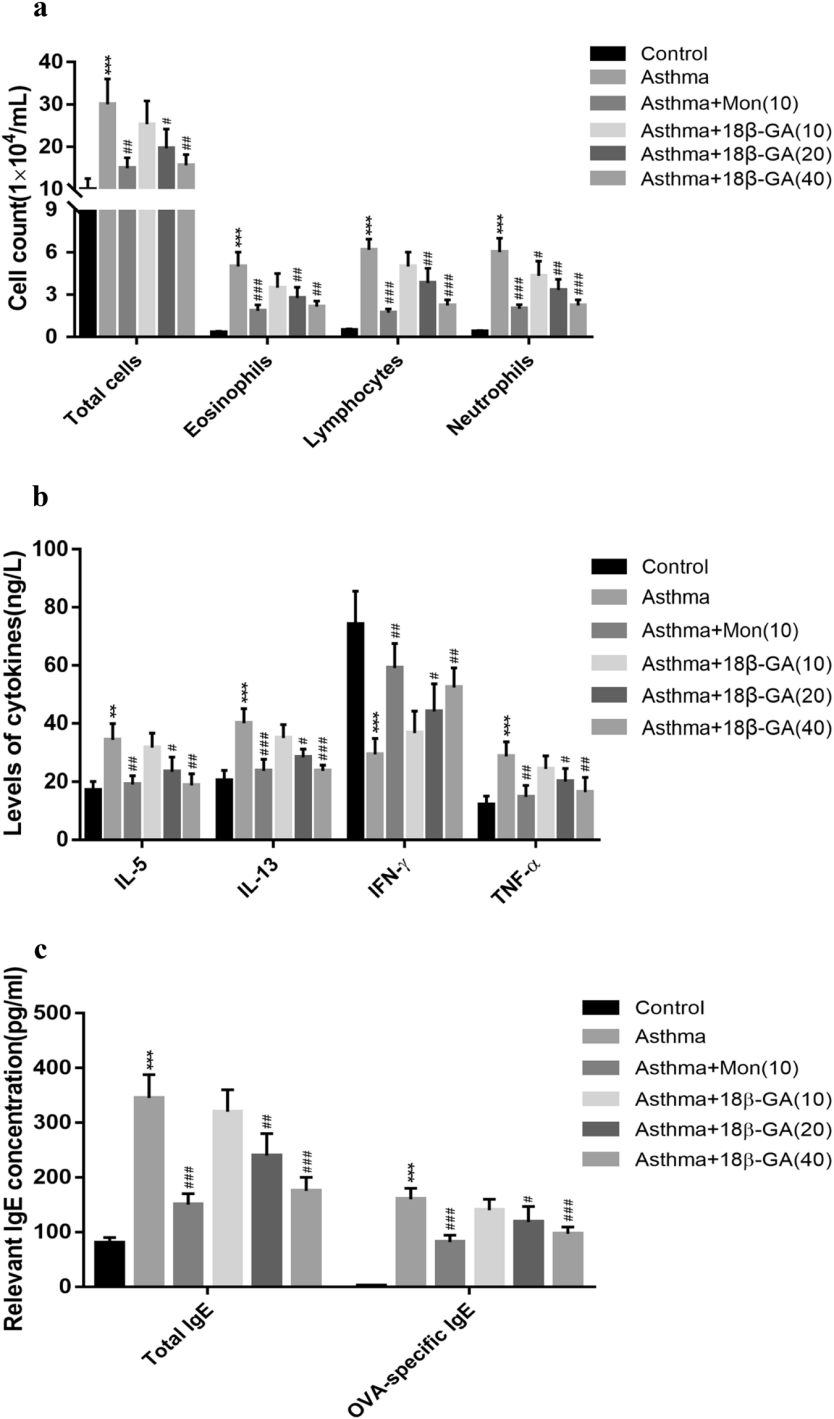
Effect of 18β-GA on the generation of inflammatory cell infiltration, IL-5, IL-13, IFN-γ, TNF-*α* and IgE in BALF. (**a**) Cell counts in BALF were measured by Diff-Quik staining. (**b**) The expression levels of IL-5, IL-13, IFN-γ and TNF-α in BALF were measured by ELISA. (**c**) The concentration of IgE in BALF was measured by ELISA. Data are shown as the mean ± SD (n = 6). *p < 0.05, **p < 0.01, ***p < 0.001 *vs*. control group, ^#^*p* < 0.05, ^##^*p* < 0.01, ^###^*p* < 0.001 *vs*. asthma group.

Compared with the normal mice, the asthma mice had significantly increased total inflammatory cell counts, eosinophils, neutrophils and lymphocytes (Fig. 5a). After treatment with Mon or 18β-GA, inflammatory cell counts decreased, and the decrease was more obvious with the increase of dose. Further analysis on IL-5, IL-13, and IFN-γ levels showed that compared with the normal control, the asthma group had higher IL-5, IL-13 and TNF-α levels and lower IFN-γ levels. Treatment with 18β-GA decreased the levels of IL-5, IL-13 and TNF-α(*p* = 0,008 *p* = 0.000 and *p* = 0.006, respectively) while increased the level of IFN-γ (*p* = 0.007) (Fig. 5b). Although the OVA-specific IgE level and the total IgE level elevated significantly (*p* = 0.000 and *p* = 0.000, respectively) in the asthma group, they decreased significantly after treatment with 18β-GA (*p* = 0.000 and *p* = 0.000, respectively) (Fig. 5c).

### 18β-GA reduced oxidative stress and increased the amount of antioxidant enzymes

**Figure 6:**
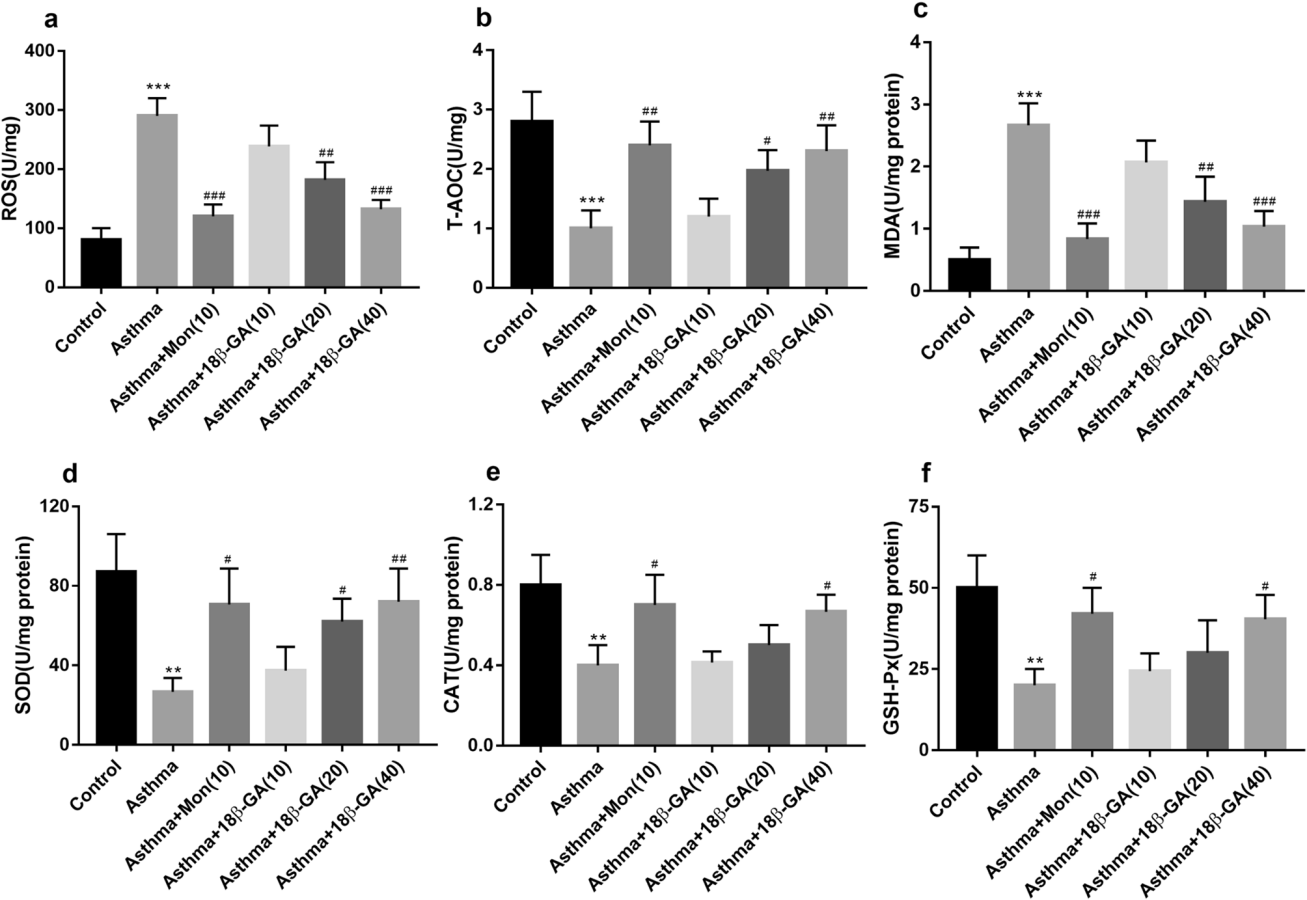
Effects of 18β-GA on oxidative stress in lung tissue. The levels of (**a**) ROS, (**b**) T-AOC, (**c**) MDA, (**d**) SOD, (**e**) CAT and (**f**) GSH-Px in lung tissues were examined. Data are shown as the mean ± SD (n = 6). *p < 0.05, **p < 0.01, ***p < 0.001 *vs*. control group, ^#^*p* < 0.05, ^##^*p* < 0.01, ^###^*p* < 0.001 *vs*. asthma group. T-AOC, total antioxidant capacity; MDA, Malondialdehyde; CAT, catalase.

In mice with OVA-induced asthma, the lung levels of ROS, MDA increased and the lung levels of T-AOC, SOD, CAT and GSH-Px decreased. Compared with the asthma group without treatment, 18β-GA treatment groups had significantly lower levels of ROS and MDA (*p* = 0.000 and *p* = 0.000, respectively), and higher levels of T-AOC, SOD, CAT, and GSH-Px (*p* = 0.006, *p* = 0.008, *p* = 0.024 and *p* = 0.031, respectively), protecting the lungs from oxidative damage (Fig. 6).

### 18β-GA inhibits allergic inflammation of airway through the NF-κB and Nrf2/HO-1 pathways

**Figure 7:**
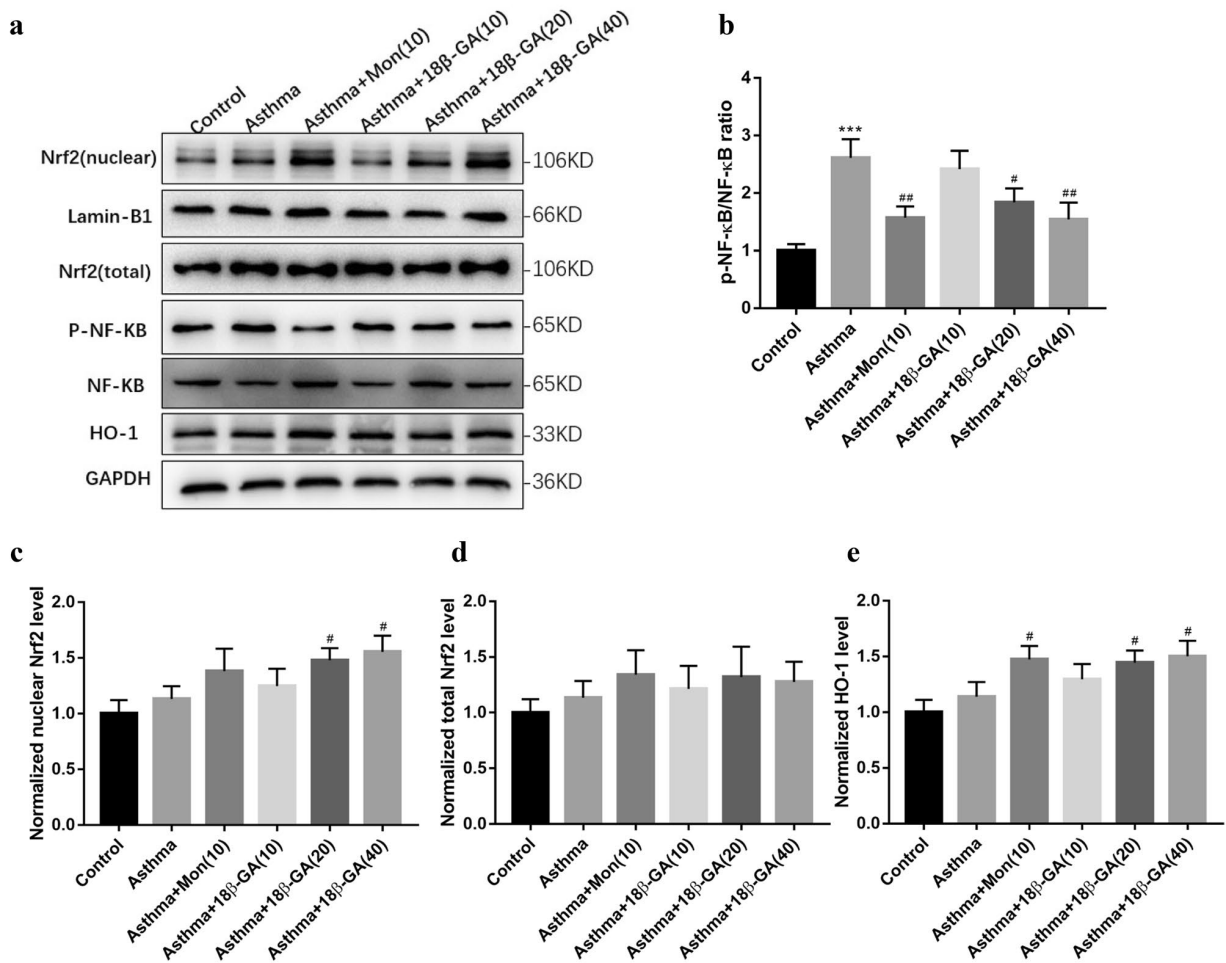
Effect of 18β-GA on inflammation and oxidative stress signaling pathway. (**a**) The protein expression of Nrf2, p-NF-κB, NF-κB and HO-1 was determined by western blotting. (**b**) The relative density quantification of NF-κB. The results were expressed as the ratio of phosphorylated proteins relative to total proteins. (**c**–**e**) The relative density quantifications of Nrf2 (nuclear), Nrf2 (total), and HO-1. Data were shown as mean ± SD (n = 6). *p < 0.05, **p < 0.01, ***p < 0.001 *vs*. control group, ^#^*p* < 0.05, ^##^*p* < 0.01, ^###^*p* < 0.001 *vs*. asthma group.

Previous results suggest that 18β-GA can improve lung function in mice with allergic airway inflammation induced by OVA. The effect of 18β-GA on the protein levels of phosphorylated NF-κB(p-NF-κB/p-p65), Nrf2 and HO-1 were examined in mice treated with Mon and 18β-GA. The results indicated that p-NF-κB, Nrf2 and HO-1 protein expressions were up-regulated in the asthma group, while p-NF-κB expression were down-regulated, while nuclear Nrf2 and HO-1 expression were further up-regulated after 18β-GA(40) treatments(*p* = 0.014 and *p* = 0.015, respectively) (Fig. 7).

### Effects of 18β-GA on pro-inflammatory cytokines and Th2 cytokines production in TNF-α-stimulated NCI-H292 cells

**Figure 8:**
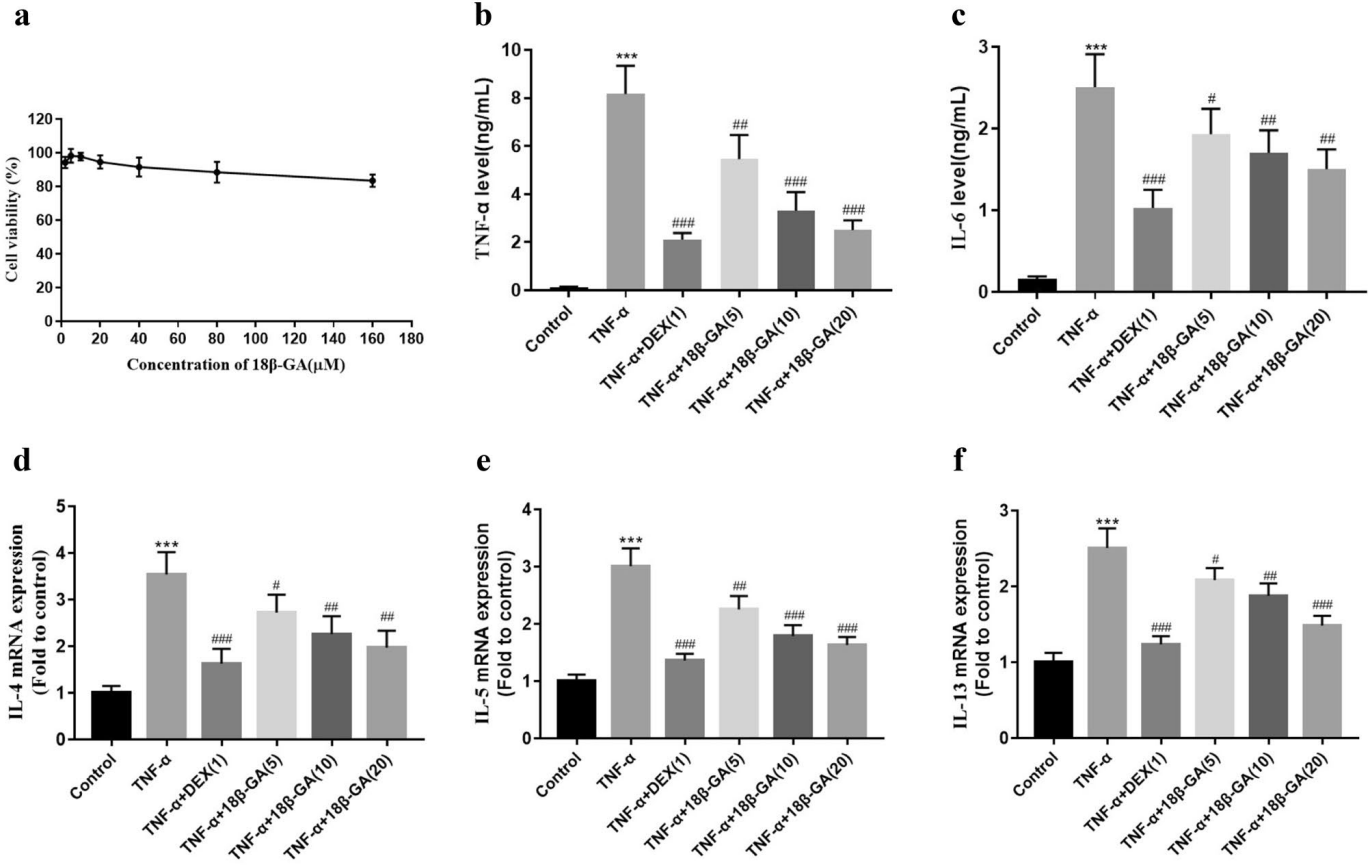
Effects of 18β-GA on cytotoxicity, pro-inflammatory cytokines and Th2 cytokines (IL-4, IL-5 and IL-13) in NCI-H292 cells. (**a**) The cytotoxicity was measured using CCK-8 kit. (**b**) The levels of TNF-α and (**c**) IL-6 were determined by ELISA kit. The mRNA levels of (**d**) IL-4, (**e**) IL-5, and (**f**) IL-13 were measured by real-time PCR in TNF-α-stimulated NCI-H292 cells. Data were shown as mean ± SD (n = 3). *p < 0.05, **p < 0.01, ***p < 0.001 *vs*. control group, ^#^*p* < 0.05, ^##^*p* < 0.01, ^###^*p* < 0.001 *vs*. TNF-α-stimulated group.

Based on the results of cytotoxic effects of 18β-GA (Fig. 8a), nontoxic concentrations (5, 10, and 20 µM) of 18β-GA were employed in next study. TNF-α-stimulated cells increased the production of TNF-α and IL-6. However, 18β-GA-treated cells had a remarkably decrease in the levels of them in a concentration-dependent manner (Fig. 8b,c). TNF-α-stimulated cells promote the expression of IL-4, IL-5 and IL-13 mRNA compared with the non-treated cells, whereas 18β-GA treatment could reduce the mRNA levels of IL-4, IL-5 and IL-13 (Fig. 8d–f).

### Effects of 18β-GA on NF-κB and Nrf-2/HO-1 pathway, ROS production and oxidaive stress markers in TNF-α-stimulated NCI-H292 cells

**Figure 9:**
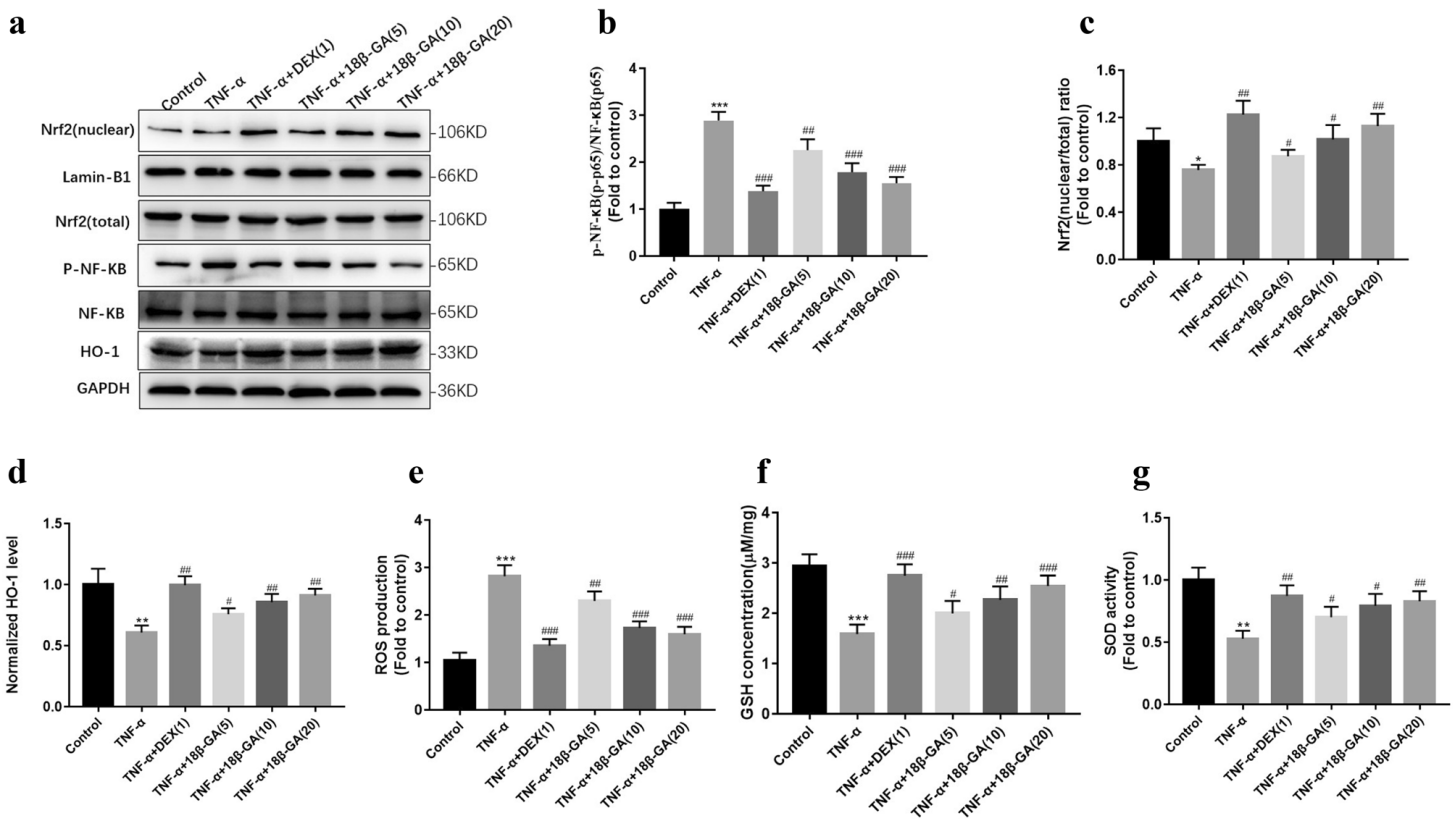
Effects of 18β-GA on NF-κB, Nrf-2, HO-1 and oxidative stress makers in NCI-H292 cells. (**a**) The p-NF-κB(p-p65), (**b**) nuclrear Nrf-2 and (**C**) HO-1 were analyzed by western blotting. The levels of (**d**) ROS and (**e**) GSH, and activities of (**f**) SOD were determined in NCI-H292 cells. Data were shown as mean ± SD (n = 3). *p < 0.05, **p < 0.01, ***p < 0.001 *vs*. control group, ^#^*p* < 0.05, ^##^*p* < 0.01, ^###^*p* < 0.001 *vs*. TNF-α-stimulated group.

TNF-α treatment increased the phosphorylation of p-NF-κB(p65) and reduced the nuclear translocation of Nrf-2 and HO-1 (Fig. 9a–d). TNF-α treatment up-regulated the ROS production and down-regulated the GSH content and SOD activity in NCI-H292 cells (Fig. 9e–g). On the contrary, 18β-GA treatment inhibited the phosphorylation of NF-κB(p65) compared with the TNF-α treated cells. And it increased the translocation of Nrf-2 into the nucleus with elevated HO-1 expression. 18β-GA suppressed ROS production, increased GSH contents and activities of SOD markedly.

## Discussion

In recent years, more and more researchers have found the effectiveness of Chinese medicine for relieving asthma in the treatment of moderate and severe allergic asthma^19–21^. Although it has been suggested that 18β-GA may reduce airway inflammation in OVA-induced asthmatic mice by inhibiting RORγt, STAT6 and Foxp3 transcriptional pathways^18^, the role of 18β-GA in antioxidant stress is still unclear. Therefore, the antiasthmatic mechanism of glycyrrhizin remains to be explored. 18β-GA treatment increased nuclear translocation of Nrf-2, HO-1 expression, and levels of GSH and SOD, and inhibited pro-inflammatory cytokines (TNF-α, IL-4, IL-5, IL-6, and IL-13) and ROS production in TNF-α-stimulated NCI-H292 cells.

Asthma is an oxidative stress disorder characterized by airway inflammation and hyperresponsiveness^22^. Cytokines contribute to the gathering of eosinophils within the lesions of the lungs^23^, facilitate the activation, maturation, and accumulation of eosinophils, and finally lead to IgE production, hyperproduction of mucus, and airway inflammation^24,25^. The regulation of pro-inflammatory cytokines is vital in the control of asthma. In the current study, 18β-GA showed protective effects in mice with OVA-induced allergic airway inflammation. The 18β-GA treatment significantly improved lung function and reduced lung inflammation including inflammatory cell infiltration. The treatment of 18β-GA reduced IL-5, IL-13, TNF-α, OVA-specific IgE level and total IgE level while increased IFN-γ level, thereby helping to reduce the influx of leukocytes, goblet cell metaplasia, and airway hyperresponsiveness, as shown by lung histopathological analysis. In addition, levels of ROS, MDA, T-AOC were examined in the current study. The 18β-GA treatment group reduced the levels of ROS and MDA significantly and restored the levels of T-AOC and SOD, CAT and GSH-Px. MDA is a ROS metabolite and its level reflects protein denaturation, lipid peroxidation, and impaired endothelial cell integrity^26,27^. During an asthma attack, the activity of peroxides, MDA, conjugated dienes increased and activity of SOD decreased significantly^28,29^. SOD, an important endogenous antioxidative enzyme in lung tissue, was restored by 18β-GA treatment, leading to relief of the oxidative damage and alleviation of asthma in our study^30^.

The signal transduction of inflammation and oxidative stress were examined to understand the anti-inflammatory and antioxidant effects of 18β-GA. NF-*κ*B is one of the main contributors to the inflammatory pathway, and NF-κB p65 phosphorylation and IκB kinase activation are characterized in respiratory tract inflammation^31,32^. Consistently, our study found that NF-κB activation was promoted by OVA exposure in asthmatic mice. In addition, NF-κB activation is related to the mechanism of ROS-mediated oxidative stress^33^. Here, the OVA treatment-induced asthma, activated the NF-κB pathway and overproduced ROS in lung tissues, which could be a result of ROS and NF-κB crosstalk. The anti-oxidative pathway Nrf2/HO-1 is vital in cellular defense. It has shown that HO-1 can relieve airway oxidative stresses, mucus hypersecretion and inflammation effectively in asthma^34^. Nrf2 is the key factor for HO-1 transcription, and in physiological conditions, it is mainly located in the cytoplasm in association with the inhibitory protein Keap1. At stimulation, Nrf2 is translocated to the nucleus, where it realizes the antioxidant effect through binding to the antioxidant response element (ARE) and activating the transcription of the HO-1 gene. In this study, 18β-GA increased the nuclear expression of Nrf2 and the lung expression of HO-1 in the OVA-treated mice significantly. The results indicate that 18β-GA reduces oxidative damage and airway inflammation. through the promotion of the Nrf2/HO-1 pathway.

In conclusion, treatment of 18β-GA on OVA-induced allergic airway inflammation can suppress eosinophilia, reduce the IgE level, pro-inflammatory cytokines level, and alleviate airway hyperresponsiveness, as evidenced by alleviation in respiratory tract inflammation and less mucus production. The anti-asthmatic potency of 18β-GA is related to the inhibition of Nrf2/HO-1 pathway activation and NF-κB phosphorylation. The study indicates that 18β-GA may be a promising therapy to suppress the development of asthma.

## Materials and methods

### Chemicals and reagents

OVA (CAS NO. 9006-59-1, Biotechnology grade), montelukast (CAS NO.151767-02-1, LC&T, purity ≥ 98%) and 18β-GA(CAS NO. 471-53-4, purity ≥ 98%) were obtained from Macklin Biochemical Co., Ltd. (Shanghai, China). Dexamethasone (DEX, purity ≥ 99%) was purchased from Beyotime Biotechnology Company Ltd. (Shanghai, China). NCI-H292 cell (the human lung epithelial cell line) was purchased from Procell Life Science & Technology Co., Ltd. (Wuhan, China).

### Mice

Female specific pathogen-free (SPF) inbred BALB/c mice(6 weeks, 20–25 g)were obtained from Hunan SJA Laboratory Animal Co.,Ltd (Changsha, China; license #: SCXK (Xiang) 2013-0006. The animals were maintained in an SPF animal house, with a regular 12 h/12 h light/dark cycle, relative humidity of 40–70%, and an average temperature of 25 °C. All experiments carried out in compliance with the ARRIVE guidelines and the guidelines of the United States National Institutes of Health (NIH). The study was approved by the Animal Care and Use Committee of the Medical College of Jiangxi Medical College.

### Asthmatic model induced by OVA and drug administration

The mouse model of asthma was induced by intraperitoneal injection of OVA (20 μg), which was mixed with aluminum hydroxide (2 mg) on day 1 and day 14. From day 18 to day 23, the mice received oral gavage of Mon (10 mg/kg) and 18β-GA (10, 20, and 40 mg/kg). The dose of 18β-GA is based on the research results of other researchers^18,35^ and our pre-experimental results (data not provided). The control group received saline of the same amount. On day 21 to day 23, 1% OVA aerosol in PBS was given to the sensitized mice for 20 min. On day 24, whole-body plethysmography (Buxco Electronics, Troy, NY) was used to evaluate airway hyperresponsiveness. There were 6 groups of mice, with 6 mice in each group (n = 6): Control (normal control), Asthma (OVA sensitization), Asthma + Mon (OVA sensitization and Mon administration), Asthma + 18β-GA 10, 20, and 40 (OVA sensitization and 18β-GA gavage of 10, 20, and 40 mg/kg, respectively). The procedure of the experiment is presented in Fig. 2.

### Measurement of airway hyperresponsiveness to inhaled MCH

The airway hyperresponsiveness was measured 24 h after the last OVA challenge. The experiment was carried out when the mice were anesthetized with sodium pentobarbital (100 mg/kg, i.p.): the mouse trachea was incised, then intubated and placed in the whole body plethysmograph, which was connected to the ventilator. Then, when the mice were exposed to increasing concentrations (0, 3.125, 6.25, and 12.5 mg/mL) of MCH, record the RL and Cdyn.

### Lung histopathology

Lung tissues were fixed with 10% formalin, paraffin-embedded, and cut into 4 µm sections, then stained with H&E to assess infiltration of inflammatory cells, or stained with PAS to assess the mucus production of goblet cells. The inflammation of the lung tissue and mucus production was quantitatively analyzed by an image analyzer.

### Assessment of bronchoalveolar lavage fluid and serum

The mice were anesthetized with sodium pentobarbital (100 mg/kg, i.p.) and the lungs were exposed by thoracotomy. The mice were intubated and intratracheally instilled with PBS twice before the bronchoalveolar lavage fluid (BALF) collection. After these procedures were completed, the mice were sacrificed by cervical dislocation. The lavage solution was then centrifuged at 4 °C and 500×*g* for 10 min. Differential cell counts were performed and stained with Diff-Quik (Solarbio, Beijing). The cytokines in the supernatant were detected and the cell precipitation was resuspended. The type and number of leukocytes were calculated by a cell counter. The levels of IL-4, IL-5, IL-13, IFN-γ, TNF-α and IgE were determined by ELISA kits (Nanjing Jiancheng Bioengineering Institute, China).

### Evaluation of lung oxidative stress

Samples of lung tissues was cut up and homogenized with RIPA cold lysis buffer for 3 min. After centrifugation (12,000×*g*, at 4 °C for 10 min), lung ROS, T-AOC, MDA, CAT, SOD and GSH-Px were measured on a 96 well plate by a commercially available kit (Beyotime Institute of Biotechnology). The activities of ROS, T-AOC, MDA, CAT, SOD, and GSH-Px were detected by the ROS, T-AOC, MDA, SOD, CAT, and GSH-Px assay kits, then the absorbance at 485 nm, 593 nm, 532 nm, 450 nm, 520 nm and 340 nm was measured by a microplate reader, respectively.

### Western blotting

The lung tissue and lysate containing protease inhibitor were homogenized, then centrifuged at 4 °C at 12,000×*g* to retain the supernatant. The 25 μL of 15-fold diluted supernatant was used for protein quantification. The loading buffer was added, and the remaining supernatant was separated by SDS-PAGE, and then transferred to PVDF membrane. The blots were cut prior to hybridisation with antibodies during blotting. Then sealed at room temperature for 2 h and incubated with the primary antibodies (1:1000) overnight at 4 °C. On the second day, the second antibody (1:15,000) was applied for 1 h, and the ECL kit was used for photodetection. The primary antibodies including anti-HO-1 Abs, anti-NF-κB Abs, anti-Nrf2 Abs and anti-phosphorylated NF-κB(p-NF-κB) Abs that were used in the study were purchased from Proteintech Technology Inc.

### Cell culture and cytotoxic effects of 18β-GA

NCI-H292 cells were incubated in RPMI 1640 media (Procell, Wuhan, China) with 10% FBS (Biological Industries) and 1% antibiotics at 37 °C in a 5% CO_2_ incubator. The cells were seeded in 96-well plates at a density of 5 × 10^4^ cells/well and incubated in RPMI1640 in the presence of different concentrations of 18β-GA (0, 2, 5, 10, 20, 40,80 and 160 µM). The cytotoxic effect of 18β-GA was evaluated by assessing cell numbers using a cell counting kit (CCK8 assay)^36^.

### Measurement of levels of pro-inflammatory cytokine production and ROS, and oxidative stress marker in cell

NCI-H292 cells (5 × 10^4^ cells/well) were seeded in 6-well plates in RPMI media, treated with 18β-GA (0, 5, 10, and 20 µM) and DEX(1 µM) for 2 h^37^, and then incubated in the presence of human recombinant TNF-α (20 ng/mL) for 24 h. The concentrations of TNF-α and IL-6 in the culture medium were quantified by ELISA kit (Nanjing Jiancheng Bioengineering Institute, China). The cells were collected and used to analyze ROS production, GSH and SOD, according to manufacturer’s instruction as described above.

### Quantitative real-time polymerase chain reaction (PCR) analysis

The cells were treated with 18β-GA (0, 5, 10, and 20 µM) and DEX (1 µM) followed by TNF-α(20 h). Then, the cells were washed with PBS and total ribonucleic acid (RNA) was extracted using an RNA extraction kit (TransGen Biotech, Beijing, China). Real-time PCR was performed in triplicate with the CFX96 Touch™ Real-time PCR detection system (Bio-Rad Laboratories). All primers were synthesized by TSINGKE(Wuhan, China). The sequences of all primers are listed in Table 1.

**Table 1:** Sequences of primers for real-time quantitative.

Gene	Forword primer(5′ → 3′)	Reverse primer(5′ → 3′)
GAPDH	CAGGAGGCATTGCTGATGAT	GAAGGCTGGGGCTCATTT
IL-4	ATGGGTCTCACCTCCCAACT	TATCGCACTTGTGTCCGTGG
IL-5	CAGGGAATAGGCACACTGGA	TCTCCGTCTTTCTCCACAC
IL-13	TGGTATGGAGCATCAACCTGAC	GCATCCTCTGGGTCTCG

### Statistical analysis

Data are expressed as mean ± standard deviation (SD). The analysis used one-way ANOVA with Tukey post hoc test. Graphs are generated using Graphpad prism 8. The statistically significances were set as the following: *p* < 0.05, *p* < 0.01 and *p* < 0.001. A *p*-value < 0.05 was considered to be statistically significant (Supplementary Information).

## Supplementary Information

Supplementary Information 1.

Supplementary Information 2.
