# Supplementary material for: 18β-Glycyrrhetinic acid suppresses allergic airway inflammation through NF-κB and Nrf2/HO-1 signaling pathways in asthma mice
Source: Sci Rep. 2022 Feb 24;12:3121. doi: 10.1038/s41598-022-06455-6 (PMC8873505; doi:10.1038/s41598-022-06455-6)
Supplement: Supplementary file 1 — Supplementary Information 1. [file 41598_2022_6455_MOESM1_ESM.pdf]

FIG.8

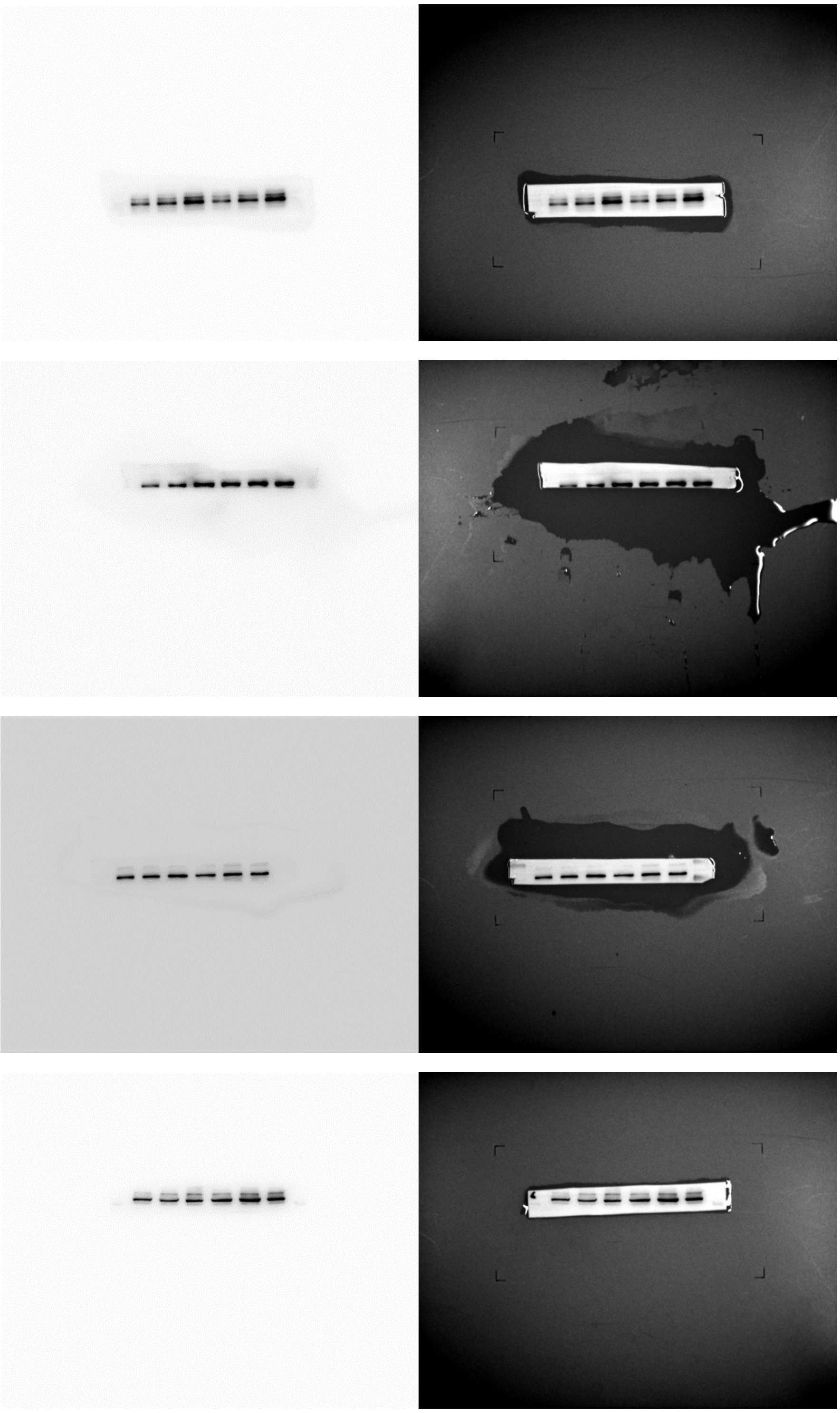

Nrf2(nuclear)1

Nrf2(nuclear)2

Nrf2(nuclear)3

Nrf2(nuclear)4

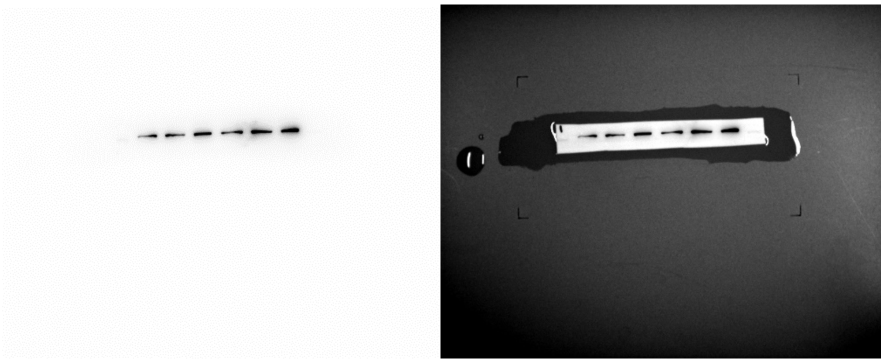

Nrf2(nuclear)5

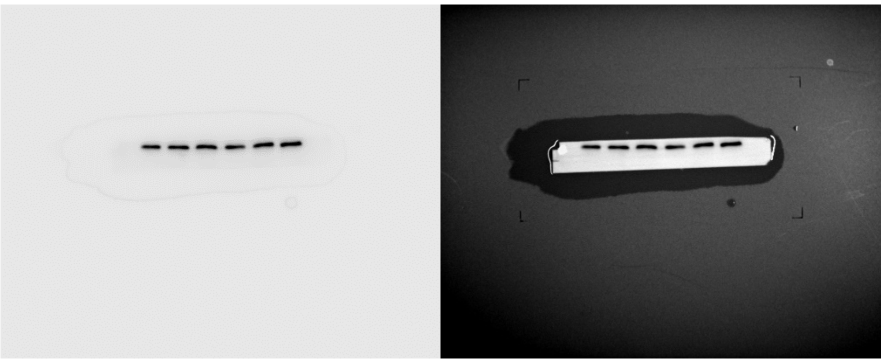

Nrf2(nuclear)6

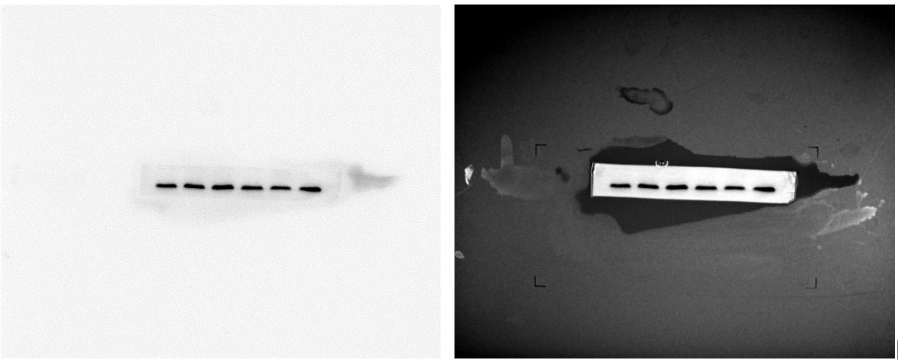

LaminB1-1

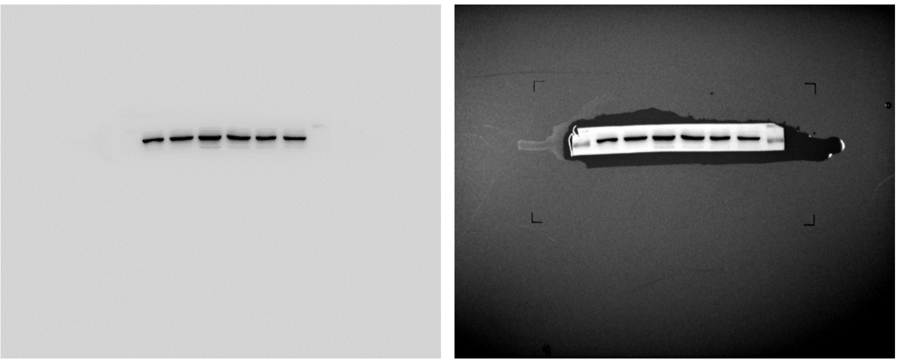

LaminB1-2

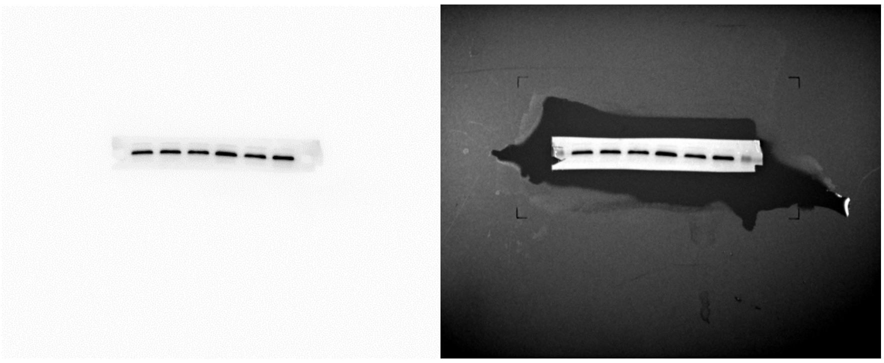

LaminB1-3

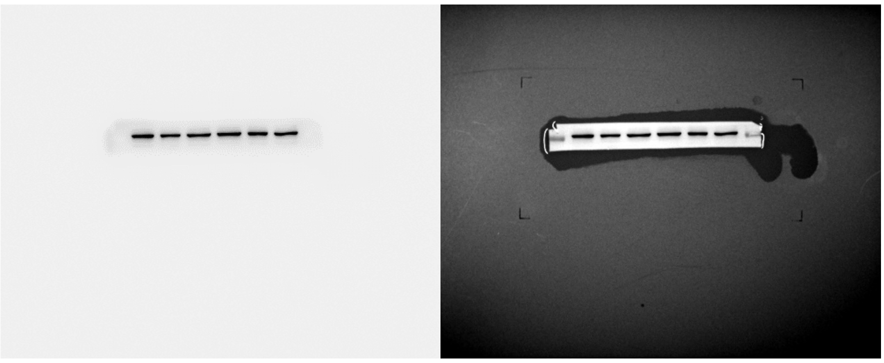

LaminB1-4

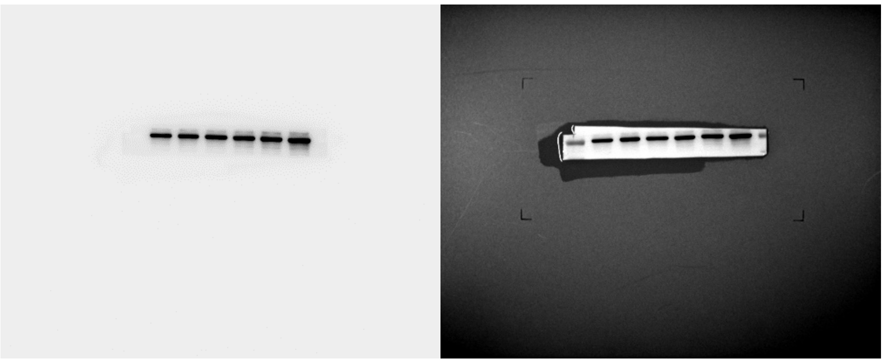

LaminB1-5

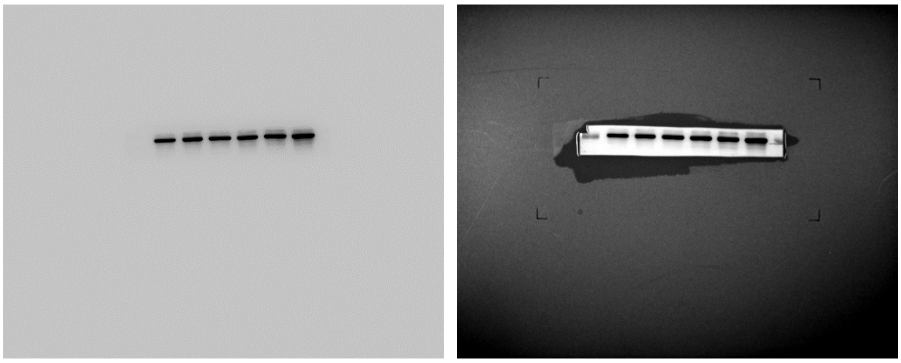

LaminB1-6

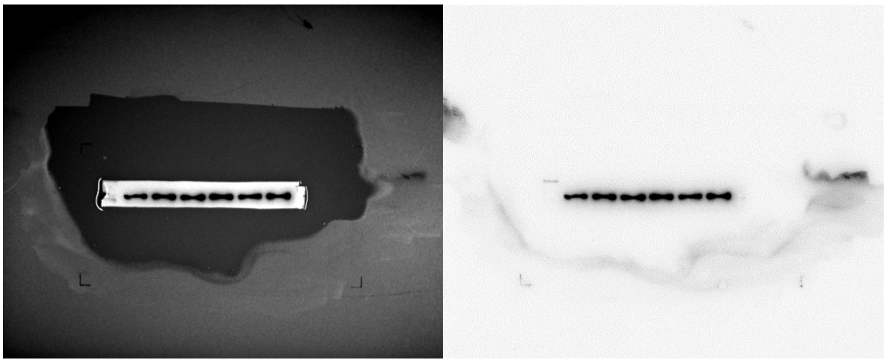

Nrf2(total)1

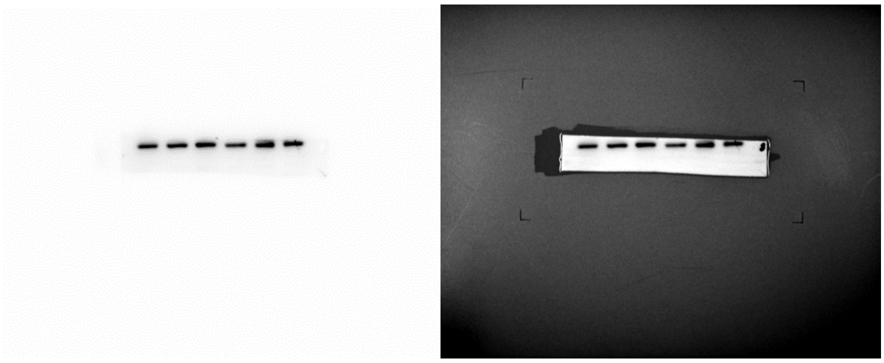

Nrf2(total)2

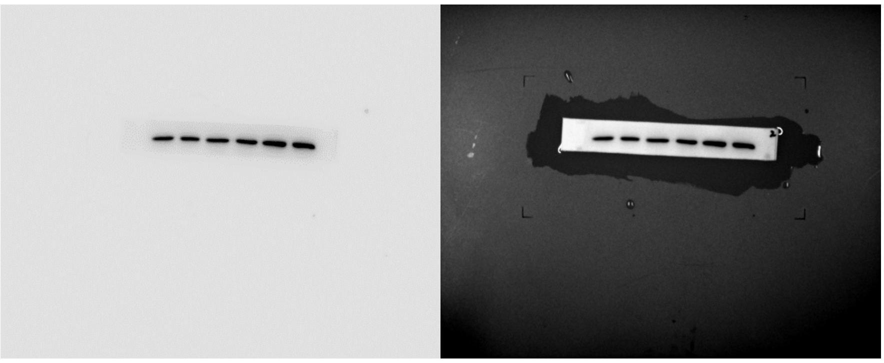

Nrf2(total)3

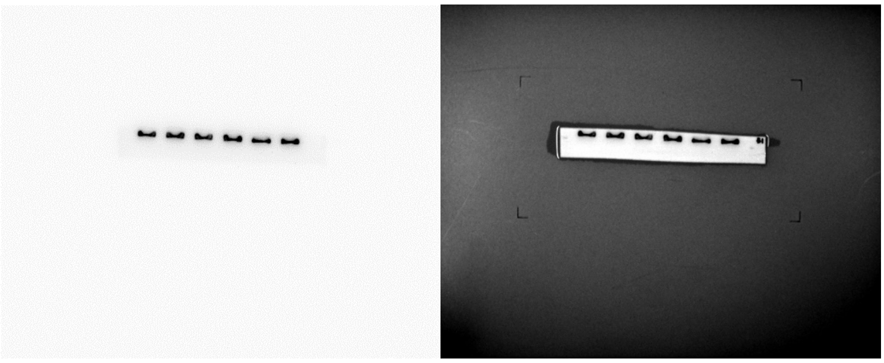

Nrf2(total)4

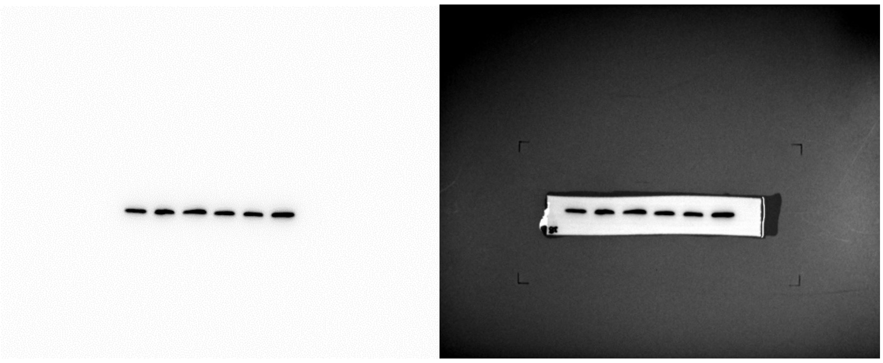

Nrf2(total)5

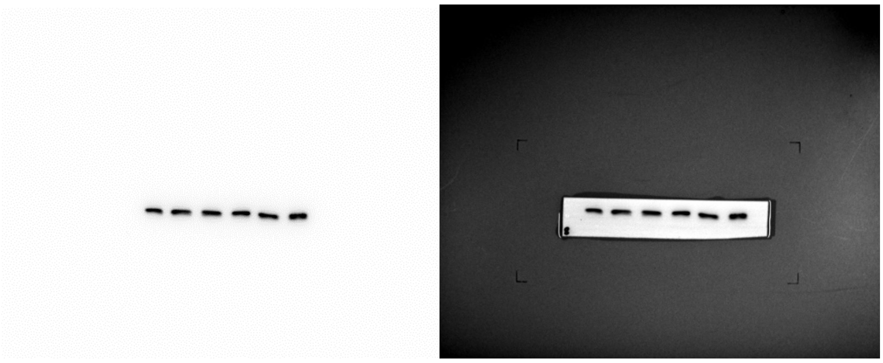

Nrf2(total)6

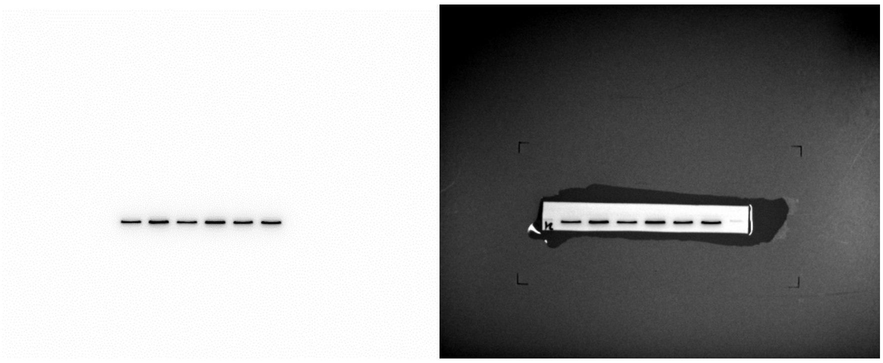

p-NF-kB1

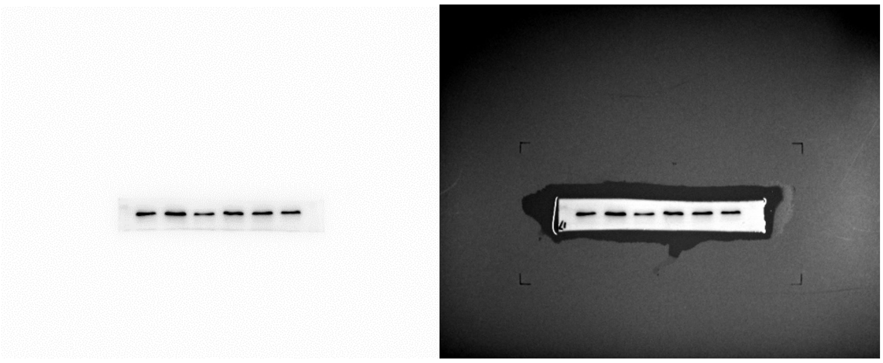

p-NF-kB2

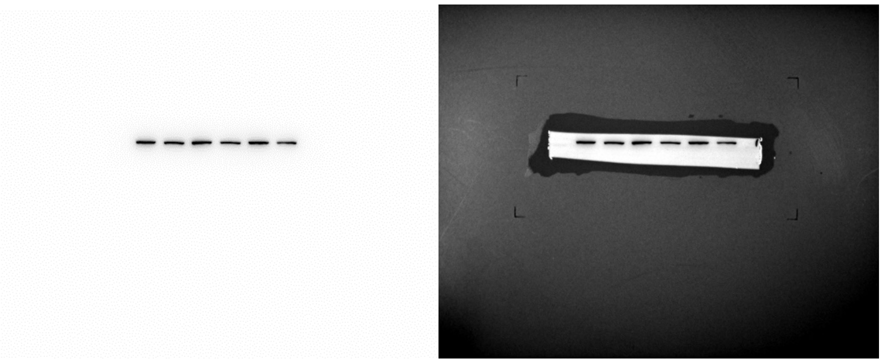

p-NF-kB3

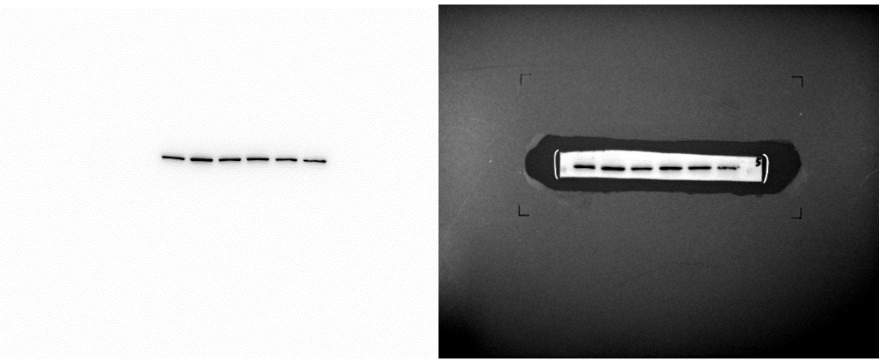

p-NF-kB4

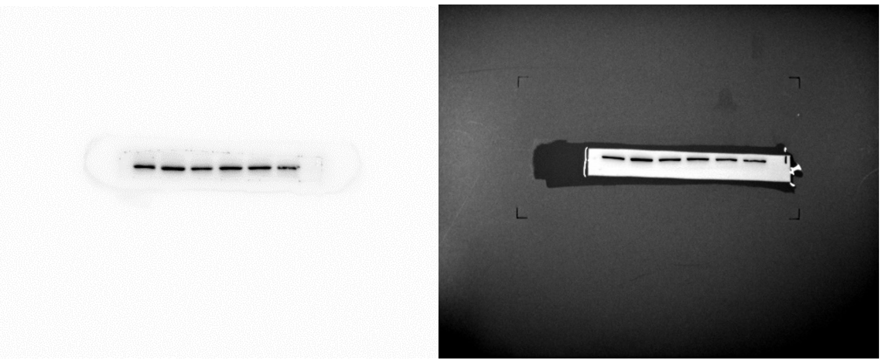

p-NF-kB5

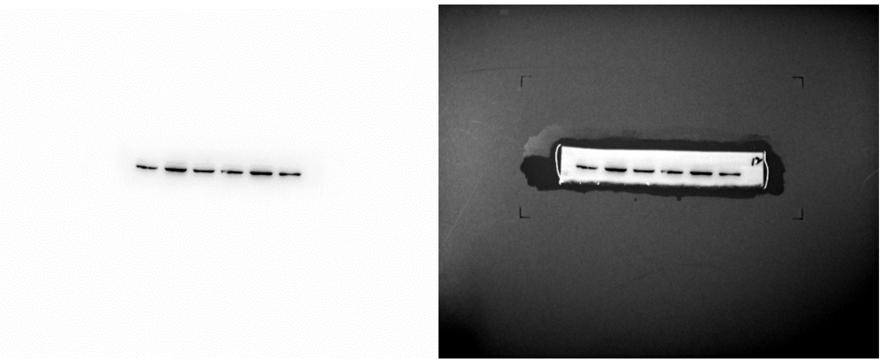

p-NF-kB6

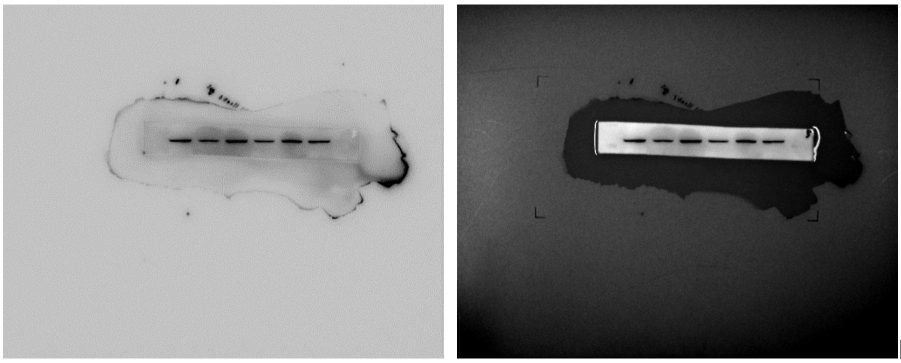

NF-KB1

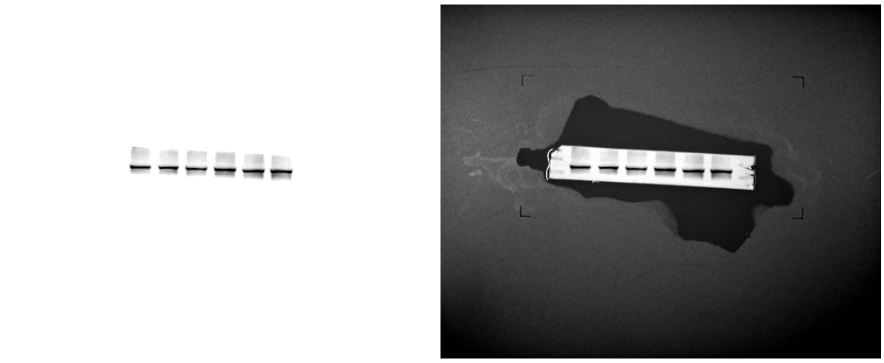

NF-KB2

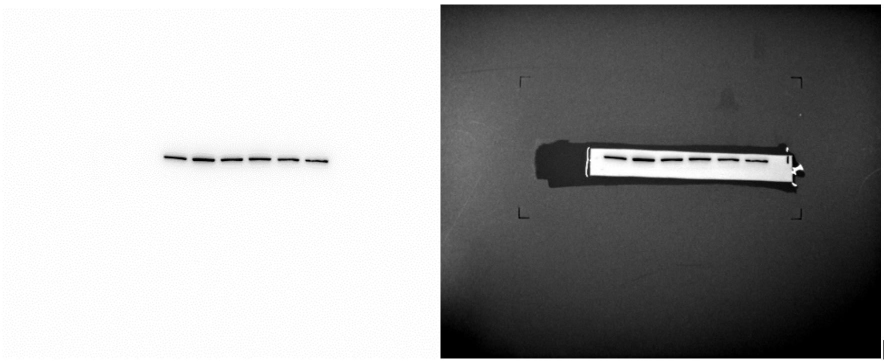

NF-KB3

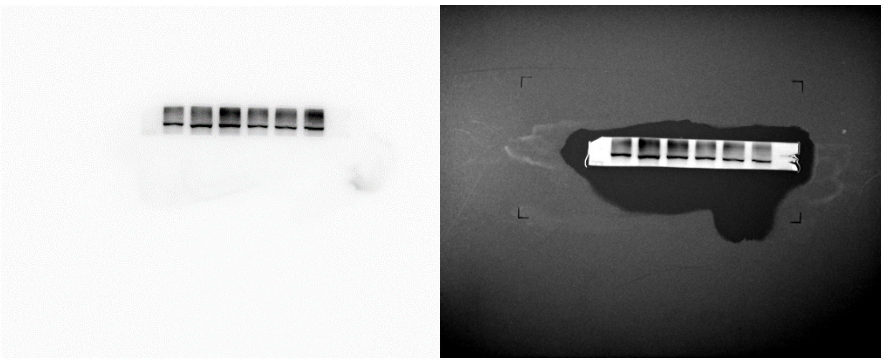

NF-KB4

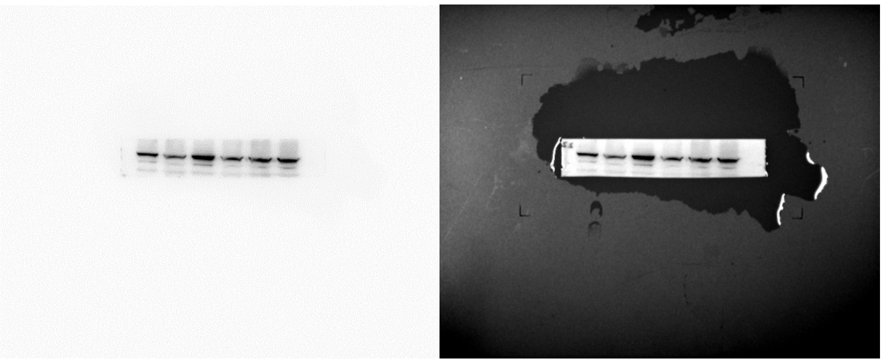

NF-KB 5

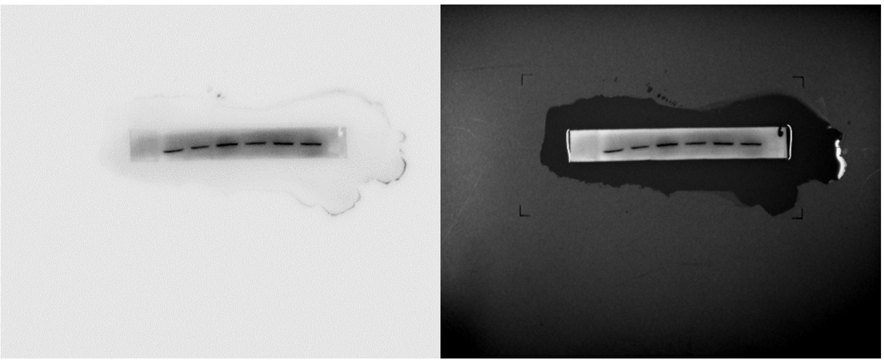

NF-KB6

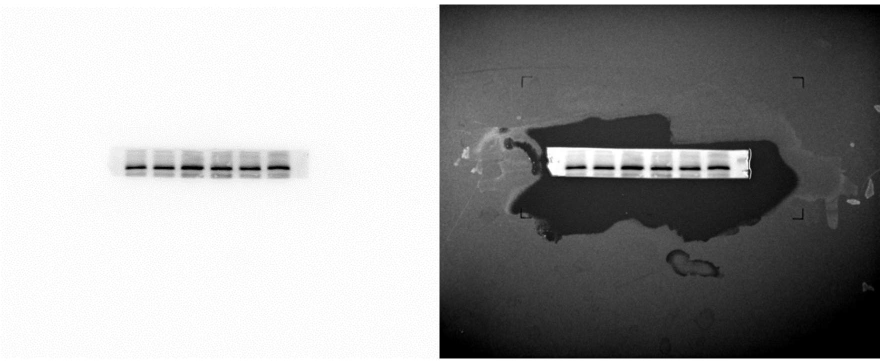

HO-1-1

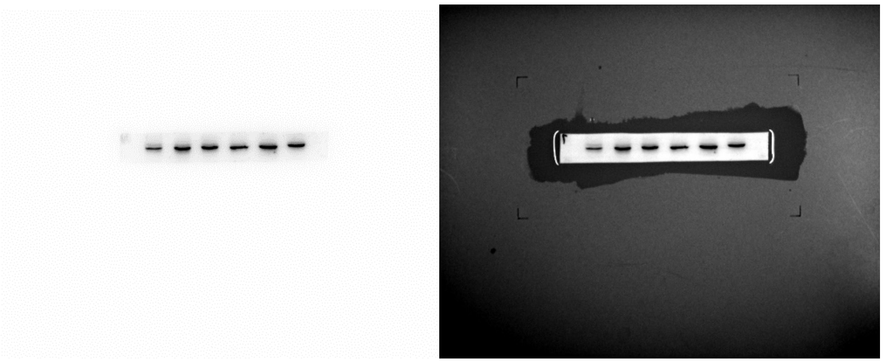

HO-1-2

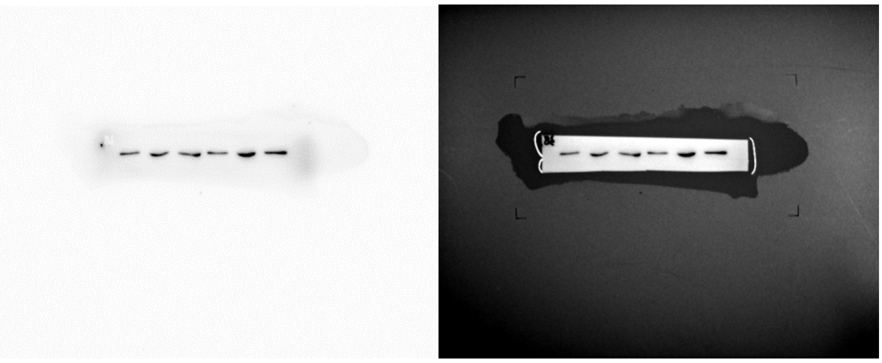

HO-1-3

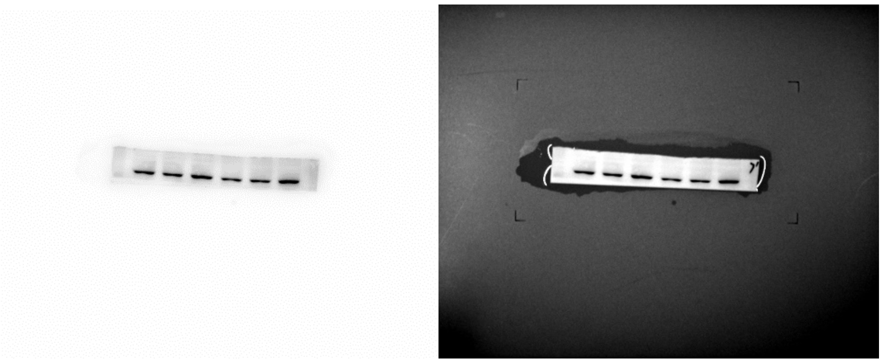

HO-1-4

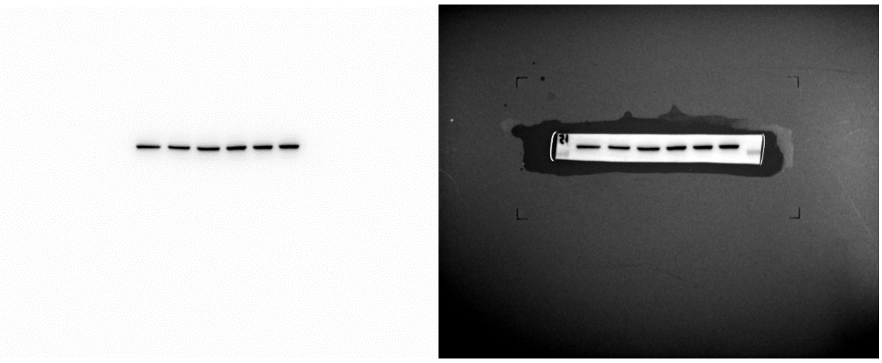

HO-1-5

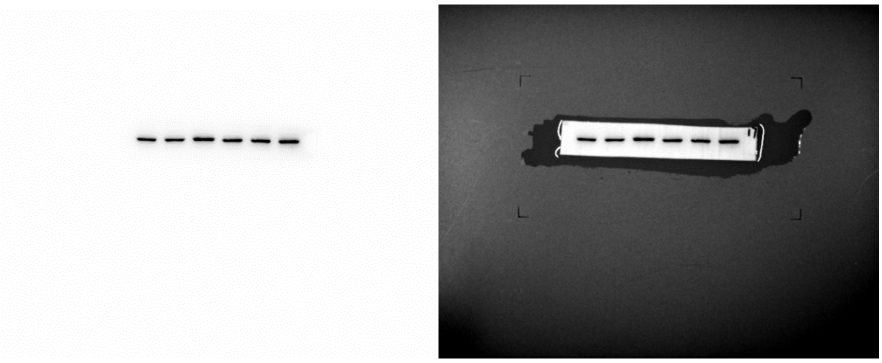

HO-1-6

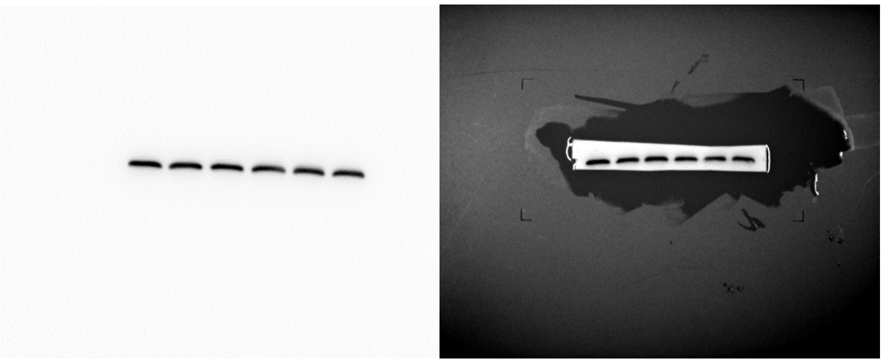

GAPDH1

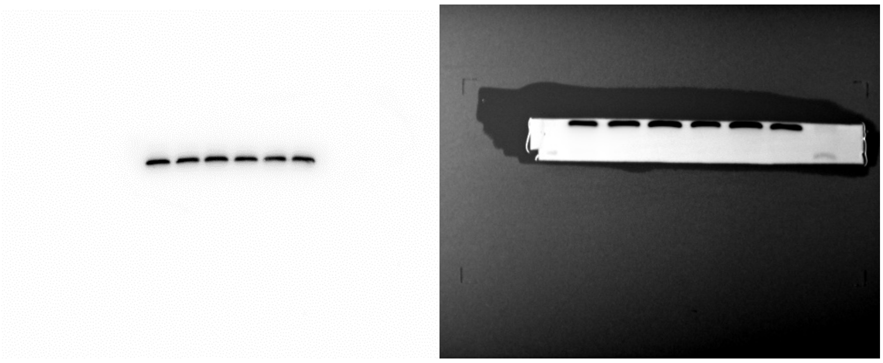

GAPDH 2

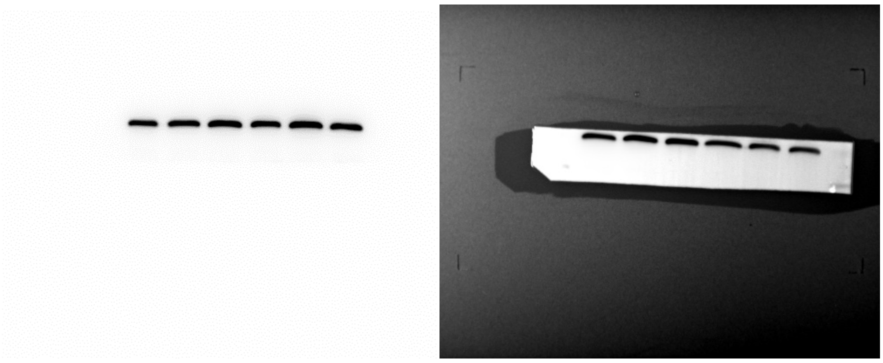

GAPDH 3

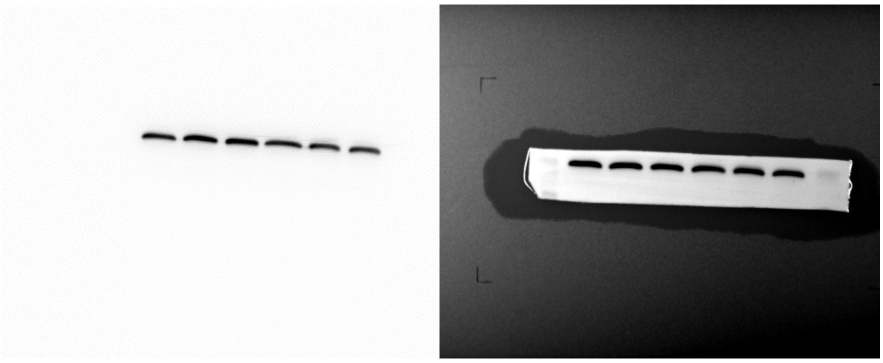

GAPDH4

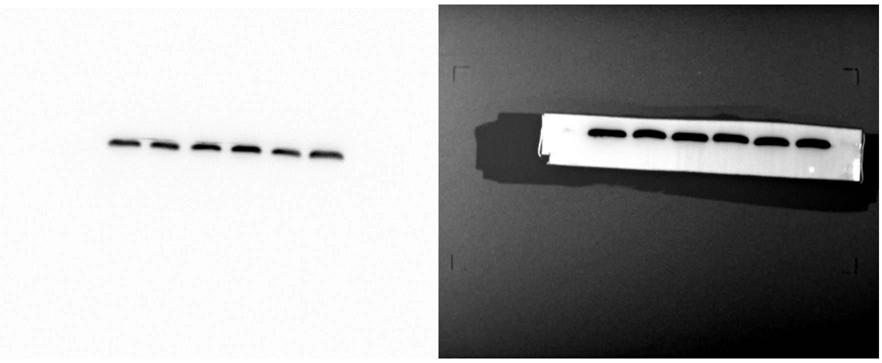

GAPDH 5

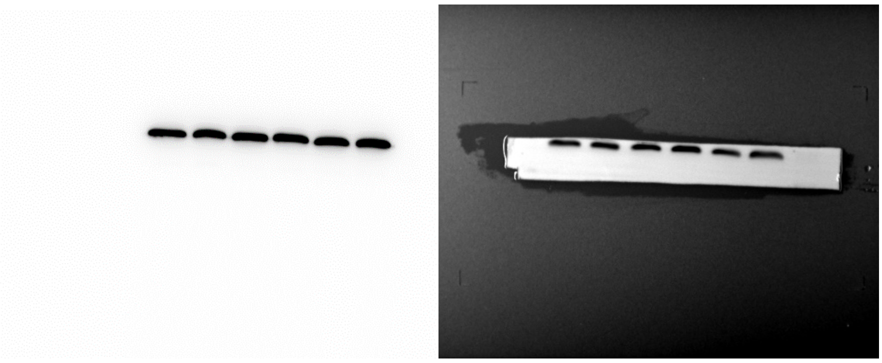

GAPDH6
