# Supplementary material for: 18β-Glycyrrhetinic acid suppresses allergic airway inflammation through NF-κB and Nrf2/HO-1 signaling pathways in asthma mice
Source: Sci Rep. 2022 Feb 24;12:3121. doi: 10.1038/s41598-022-06455-6 (PMC8873505; doi:10.1038/s41598-022-06455-6)
Supplement: Supplementary file 2 — Supplementary Information 2. [file 41598_2022_6455_MOESM2_ESM.pdf]

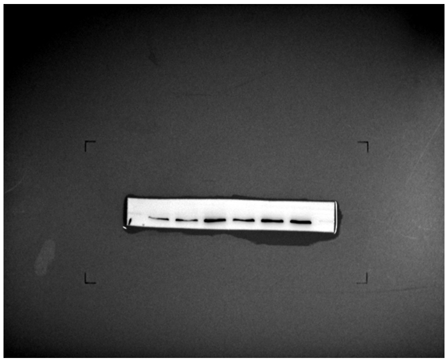

Nrf2(nuclear)1

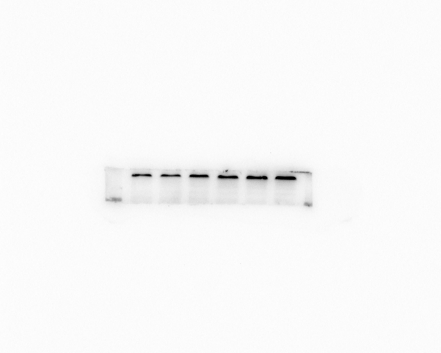
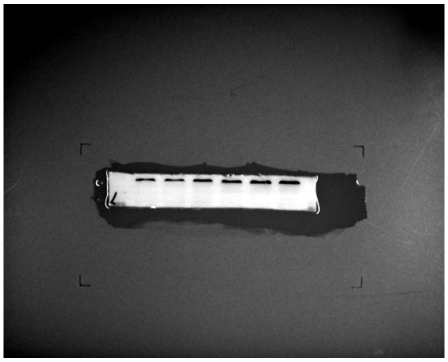

Nrf2(nuclear)2

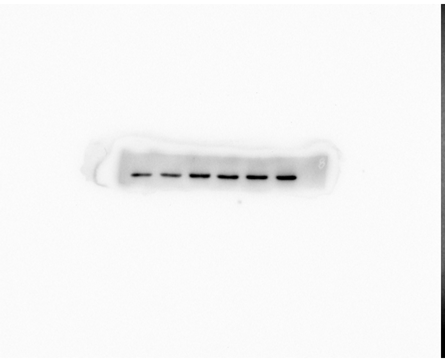
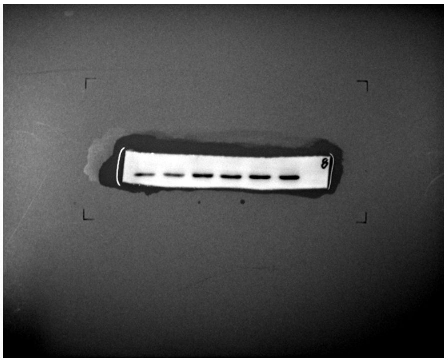

Nrf2(nuclear)3

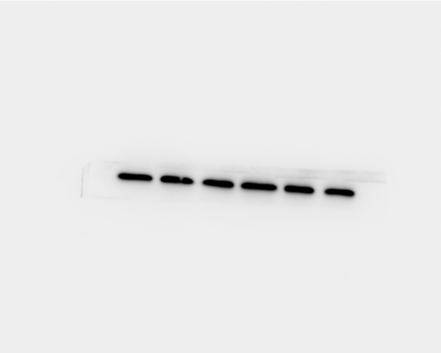
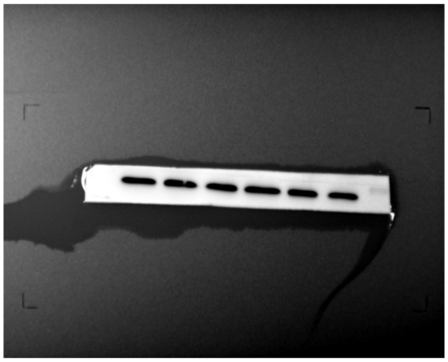

LaminB1-1

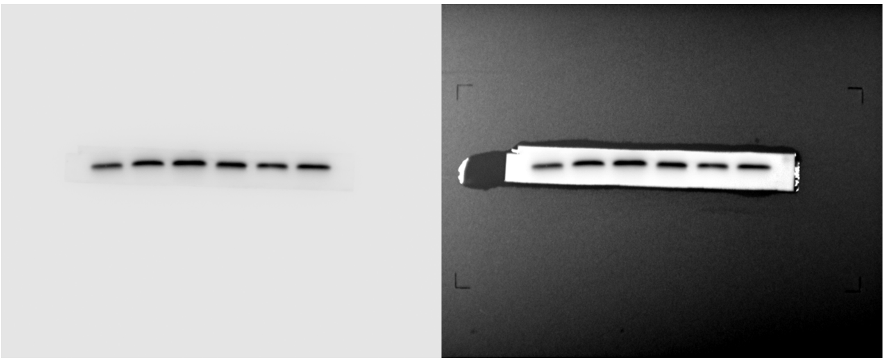

LaminB1-2

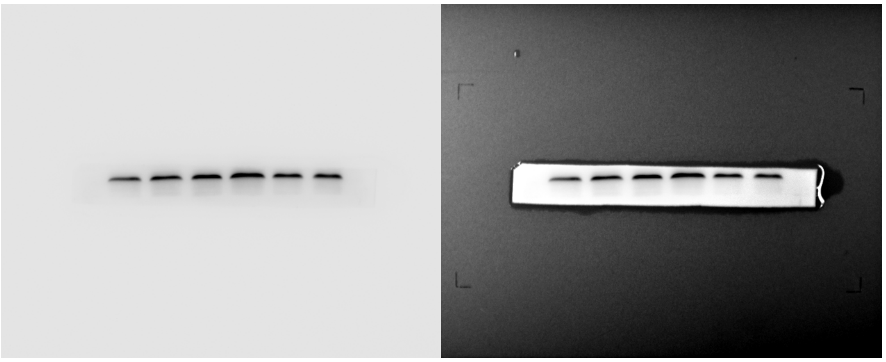

LaminB1-3

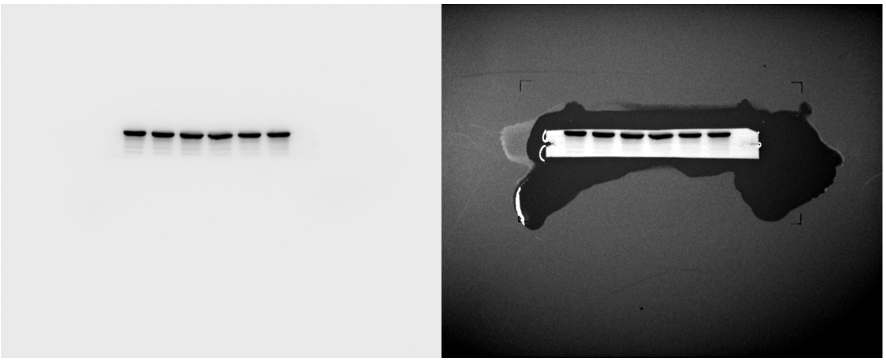

Nrf2(total)1

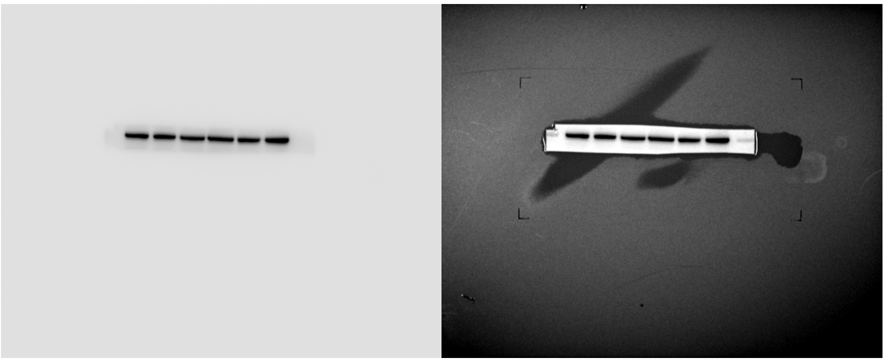

Nrf2(total)2

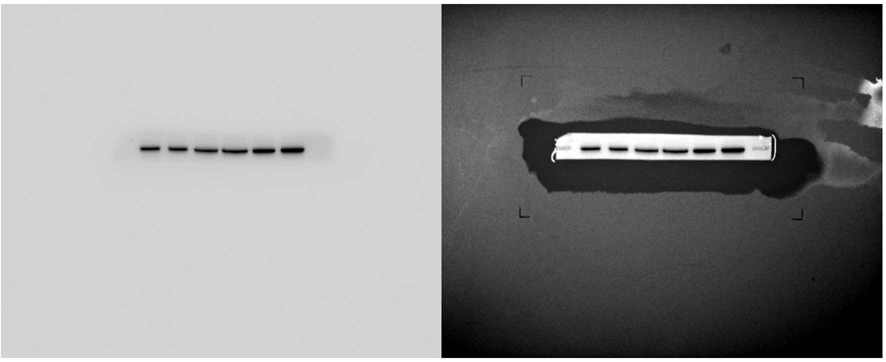
Nrf2(total)3

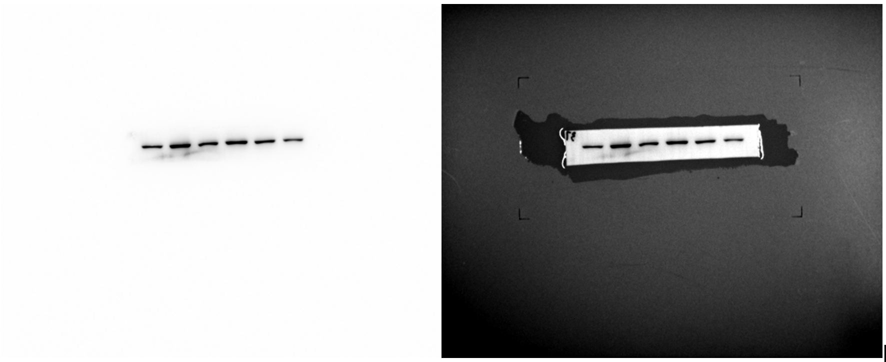
p-NF-KB1

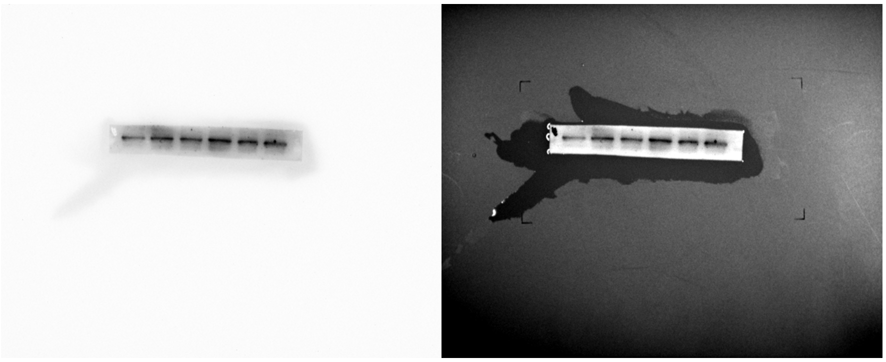
p-NF-KB2

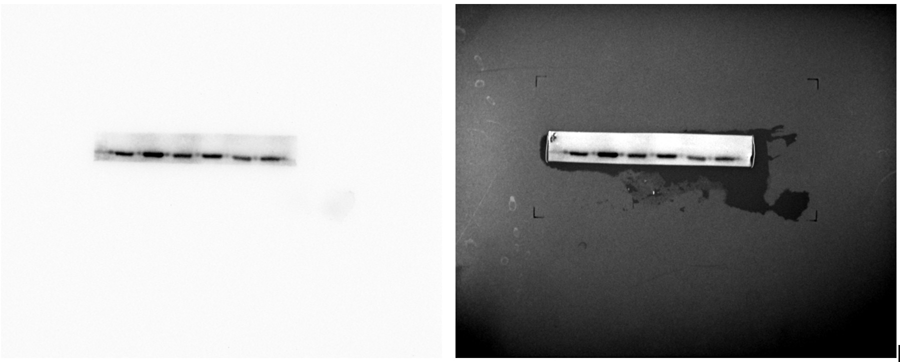
p-NF-KB3

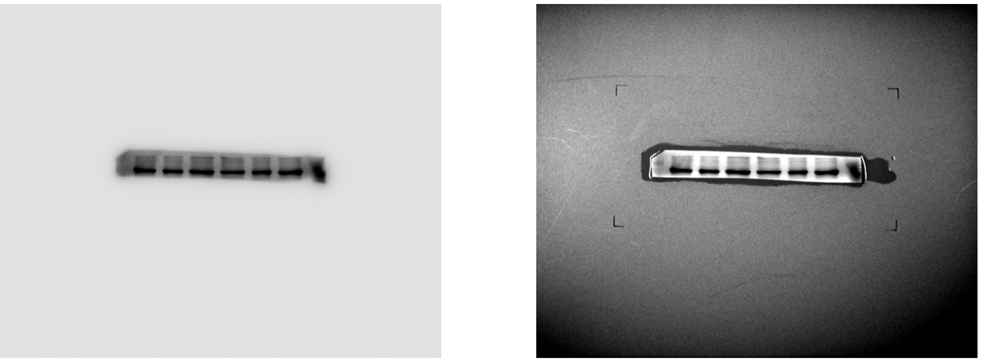

NFKB1

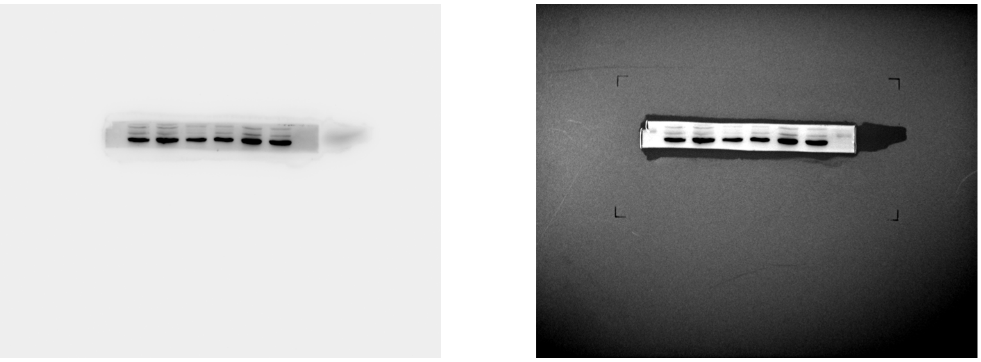

NFKB2

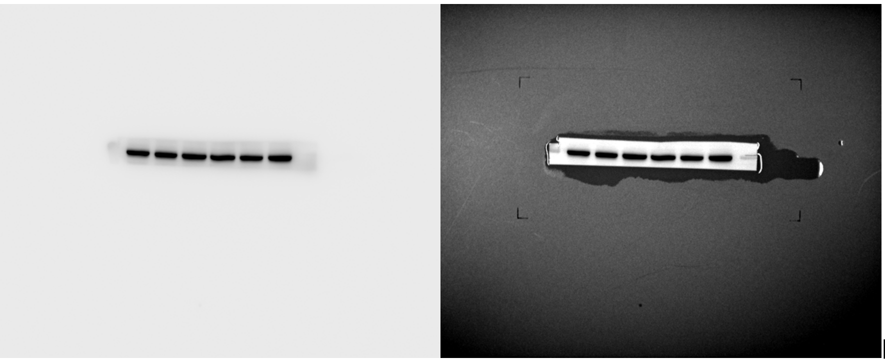

NFKB3

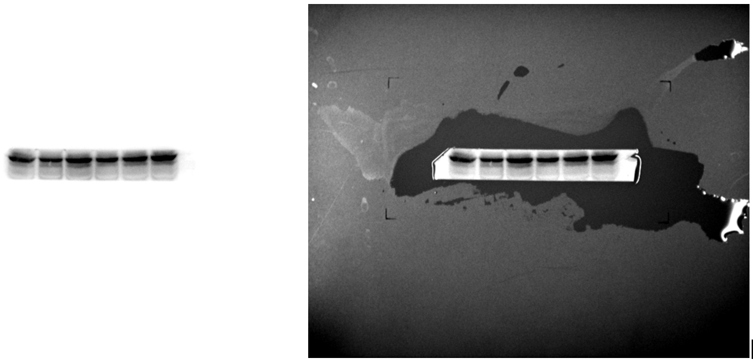

HO-1

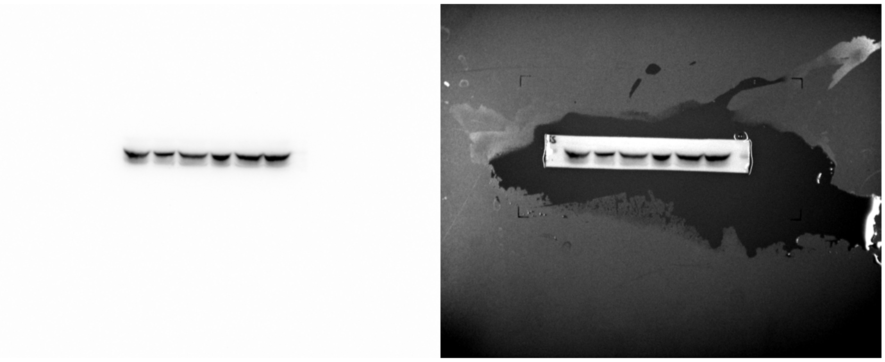

HO-2

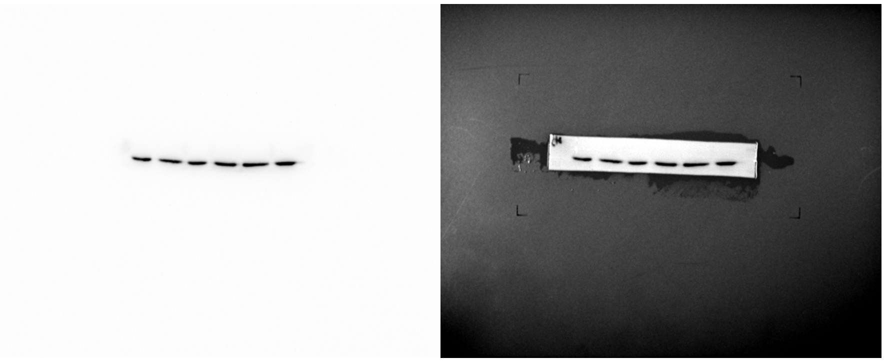

HO-3

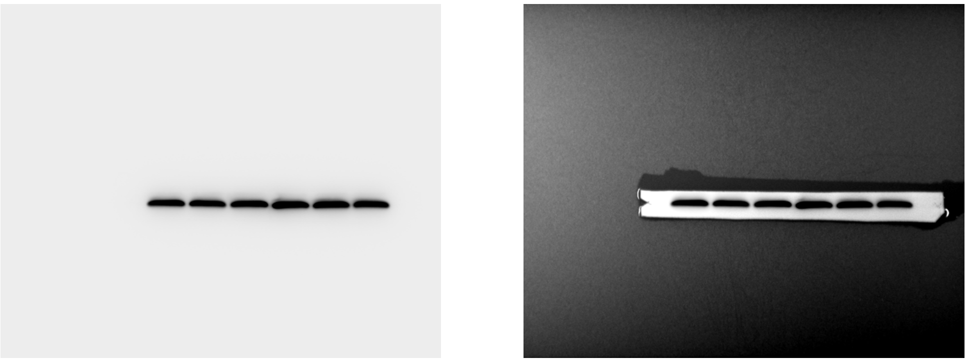

GAPDH1

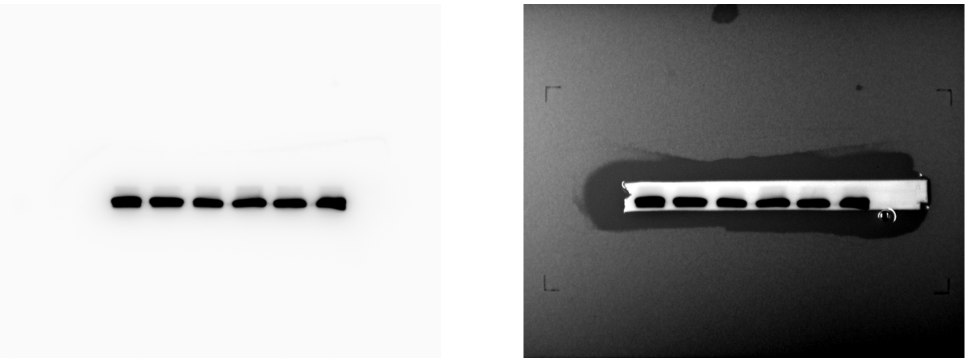

GAPDH2

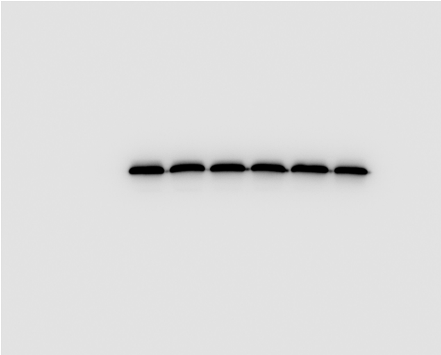
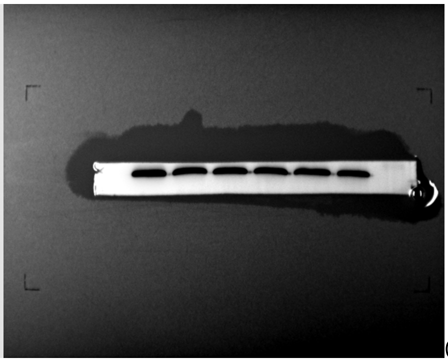
GAPDH3
